# The Nitrogen Preference of Cactus Pear (*Opuntia ficus-indica*): A Sand Culture Snapshot

**DOI:** 10.3390/plants13243489

**Published:** 2024-12-13

**Authors:** Nicholas A. Niechayev, Paula N. Pereira, John C. Cushman

**Affiliations:** 1 D’Arrigo California, 21777 Harris Rd, Salinas, CA 93908, USA; nniechay@gmail.com; 2 Hillsboro Innovative Therapies (HIT), Genentech, Hillsboro, OR 97124, USA; paula.bio.ufscar@gmail.com; 3 Department of Biochemistry and Molecular Biology, University of Nevada, Reno, NV 89557, USA

**Keywords:** ammonium, crassulacean acid metabolism, nitrate, soluble sugars, starch, tissue acidity

## Abstract

Cactus pear (*Opuntia-ficus indica* (L.) Mill.) is an important agricultural crassulacean acid metabolism (CAM) species used as a source of food, forage, fodder, and secondary products and as a biofuel feedstock. However, the preferred source of nitrogen for this species, whether it be nitrate (NO_3_^−^), ammonium (NH_4_^+^), or a combination of both, is not well understood. To investigate the nitrate and ammonium preference of cactus pear, we grew cladodes in sand culture with deionized water as a control or with a cross-factorial set of nutrient solutions of 0.0, 2.5, 5.0, and 10.0 mmol of nitrate and/or ammonium for one month. We then assessed a set of physiological parameters including cladode growth, relative water content, chlorophyll, tissue acidity, soluble sugars, starch, nitrate, ammonium, glyoxylic acid, nitrate reductase activity, and nitrogen and carbon content. We found significant differences in all measured parameters except for cladode length, relative water content, and carbon content. Cladodes provided with only deionized water produced no new cladodes and showed decreased soluble sugar content, increased starch content, and increased tissue acidity. We also determined the relative steady-state transcript abundance of genes that encode enzymes involved in N metabolism and CAM. Compared with control cladodes, nutrient-supplied cladodes generally showed increased or variable steady-state mRNA expression of selected CAM-related genes and nitrogen-metabolism-related genes. Our results suggest that *O. ficus-indica* prefers fertilizers containing either equal concentrations nitrate and ammonium or more nitrate than ammonium.

## 1. Introduction

While nitrogen (N) metabolism is well studied in C_3_ and C_4_ photosynthesis species, a robust understanding of N metabolism in CAM plants is lacking [1]. Plants can take up N in the form of inorganic nitrate (NO_3_^−^) and ammonium (NH_4_^+^) [2], and in organic N forms such as urea (CO(NH_2_)_2_) and released biological matter, which is often decomposed into ammonium by microbial communities in the soil [3,4]. Nitrate and ammonium interact with one another within the soil and can limit or enhance total N uptake in plants depending upon the abundance and ratio of these two oppositely charged molecules [2]. In addition, soils containing too much nitrate or ammonium can alter plant cellular pH, which causes detrimental changes in basic cellular functions such as osmosis, diffusion, membrane stability, and enzyme activities [5]. In most standard nutrient solutions, nitrate and ammonium are present in the millimolar range in a 1:1 ratio for ionic charge balance, or with more nitrate than ammonium [6,7] as many ammonium-sensitive plant species exist [8]. The optimal nitrate and ammonium concentrations for any given plant species are dictated by adaptation to a specific environment [9,10]. Within dry, nitrate-rich landscapes, plants tend to prefer nitrate, whereas within wet, ammonium-rich landscapes, plants tend to prefer ammonium. Some species might adapt to changes in nitrate and ammonium availability in the soil within only a few generations, although the rate at which plants can adapt to new nitrogen sources appears to vary among domesticated crop species [11]. Such adaptation was shown to be limited in wild African grass species [10].

In CAM plants, inorganic nitrate and ammonium can be assimilated into roots directly by enzymes and then transported to mesophyll cells for fixation into amino acids [1]. In CAM plants, atmospheric CO_2_ is converted into bicarbonate by carbonic anhydrase (CA). Bicarbonate is then combined with phosphoenolpyruvate by phosphoenolpyruvate carboxylase (PEPC) to form oxaloacetate. PEPC is also closely linked to nitrogen metabolism in that it provides carbon structures necessary for amino acid synthesis [12], and PEPC concentrations have been shown to fluctuate with N availability [1]. Root uptake is regulated by ammonium transporters (AMTs) and nitrate transporters (NRTs). Nitrate is reduced to nitrite (NO_2_^−^) by nitrate reductase (NR) with ferredoxin (Fdx) or NADH-reducing power in roots or shoots, respectively. Nitrite is highly oxidized and needs to be transported and/or reduced to ammonia (NH_3_) by nitrite reductase (NiR) with Fdx in the stroma of shoot cells or NADH-reducing power in the stroma of root cells. Glutamine synthetase (GS) combines ammonium with an acyl phosphate intermediate of glutamate into glutamine in the cytosol and chloroplasts. Glutamine and 2-glutarate are then converted to two molecules of glutamate by glutamine oxoglutarate aminotransferase (GOGAT). Alternatively, ammonium can be converted to carbamoyl phosphate by carbamoyl phosphate synthetase which is ATP-dependent. In the mitochondria, NAD-glutamate dehydrogenase (NAD-GDH) can convert NH_4_^+^ directly into glutamate by combining with 2-oxogluterate and using NAD(P)H-reducing power. Glutamine or ammonium and aspartate can be converted into asparagine by asparagine synthetase (AS).

Several studies have documented the productivity of *O. ficus-indica* under different fertilizer treatments in the field [13,14,15,16]. Commercial N input is typically between 50 and 300 kg ha^−1^ year^−1^ depending upon *Opuntia* spp. accessions and planting densities [17,18,19]. However, these studies were conducted with a wide variety of N sources, soil types, and production goals (e.g., fruit, cladodes, seeds, and methane production) and did not reveal the nitrate and ammonium preferences of *O. ficus-indica*.

In more controlled studies, a 4-fold increase in nocturnal acidity in *O. ficus-indica* was observed when chlorenchyma N% increased 3-fold, and a higher chlorophyll content was observed in seedlings grown in concentrated Hoagland’s solution over six months [20]. A nutrient index was developed to estimate productivity given different nutrient availabilities for cacti and agave species [16], but this model was not species specific and did not specify between nitrate and ammonium for N input. Few studies have reported on the nitrate vs. ammonium preference of *O. ficus-indica* [21]. Through measuring nitrate and ammonium depletion in hydroponic solution, the plants initially took up more N when given ammonium than nitrate after 5, 10, and 15 days of treatment, but not after 20 days [21]. Furthermore, plants accumulated significantly more biomass in the above ground tissue and slightly more on average in root tissue when plants were given nitrate. The authors concluded that in hydroponic conditions, *O. ficus-indica* absorbed more N when supplied with nitrate than with ammonium, and when supplied with nitrate, the plants showed increased biomass production.

In this study, we examined the response of *O. ficus-indica* to varying amounts of nitrate vs. ammonium and combinations of these nutrients in a sand culture experiment. The hypothesis to be tested was that *O. ficus-indica* cladodes would show a preference for N inputs when provided with either nitrate or ammonium or a combination of these two forms of nitrogen. Our results suggest that *O. ficus-indica* responds to differences in nitrate and ammonium availability with a preference for fertilizers that contain either equal parts nitrate and ammonium or more nitrate than ammonium.

## 2. Results

### 2.1. Growth Measurements and Relative Water Content

**Figure 1:**
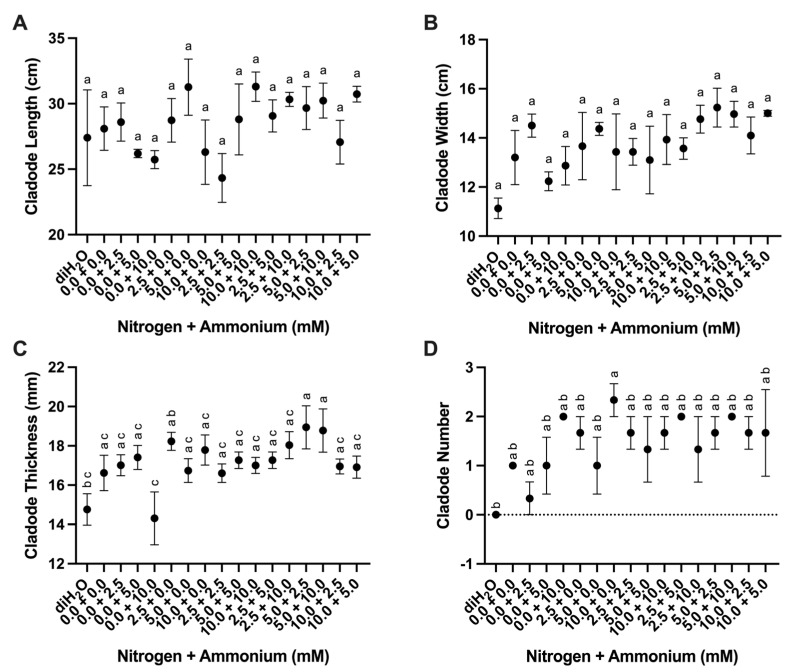
Cladode responses to nutrient treatments. (**A**) Cladode length (cm) among treatments (*n* = 3). (**B**) Cladode width (cm) among treatments (*n* = 3). (**C**) Cladode thickness (mm) among treatments (*n* = 6). (**D**) Cladode number among treatments (*n* = 3). Treatments consisted of modified Hoagland’s solution with varying amounts of nitrate and ammonium (mM) and a deionized water treatment (diH_2_O) control. Plots show the mean values with error bars indicating ± standard error of the mean (SEM). Letters represent the result of Tukey’s multiple comparisons test (α = 0.05).

We developed a factorial matrix of 16 different nutrient treatments that varied in nitrate and ammonium concentrations with modified Hoagland’s nutrient solution plus a dionized H_2_O control treatment in a sand culture system of *O. ficus-indica* cladodes growing in sand culture under greenhouse conditions (Table 1). Cladode dimensions were evaluated for changes in response to these different nutrient treatments after one month. No statistical differences were found in cladode length (Table 2, Figure 1A). However, significant differences in cladode width and thickness were observed among analyses (Table 2). The diH_2_O treatment showed the lowest average widths, whereas the 5.0 + 2.5 (mM nitrate + ammonium) and 10.0 + 5.0 treatments showed the highest average widths (Figure 1B). The lowest mean cladode thickness was observed for the diH_2_O and 0.0 + 10.0 treatments, whereas the 5.0 + 2.5 and 5.0 + 10.0 treatments showed the largest mean cladode thickness (Figure 1C). Differences in the mean number of new cladodes were highly significant among the treatments (Table 2). The diH_2_O control showed no new cladodes (Figure 1D). However, all nitrate treatments (with the exception of the 5.0 + 0.0 treatment) showed a mean number of new cladodes above 1.

The 10.0 + 0.0 treatment showed the highest number of new cladodes (Figure 1D). Mean cladode relative water content ranged from 67.8% to 77.1% among the treatments, and no significant differences were observed (Table 2, Figure S1).

**Figure 2:**
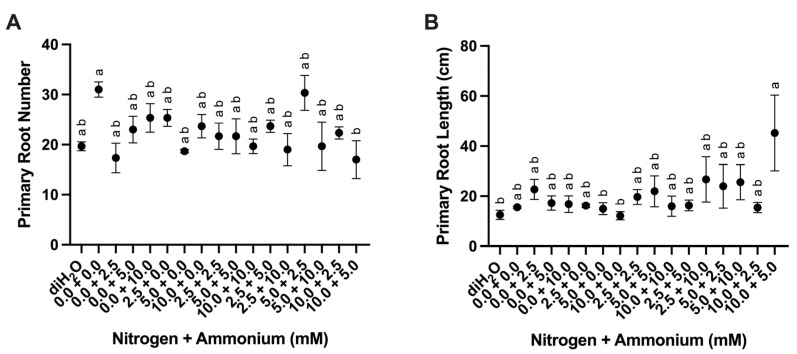
Root responses to nutrient treatments. (**A**) Primary root number among treatments (*n* = 3). (**B**) Primary root length (cm) among treatments (*n* = 3). Treatments consisted of modified Hoagland’s solution with varying amounts of nitrate and ammonium (mM) and a deionized water treatment (diH_2_O) control. Plots show the mean values ± SEM. Letters represent the result of Tukey’s multiple comparisons test (α = 0.05).

Primary mean root number was highly significantly different among analyses, and overall mean root length was also significantly different among treatments (Table 2, Figure 2A). Treatments 0.0 + 0.0 and 5.0 + 2.5 showed the highest mean root number (Figure 2A). Mean primary root length was lowest in the diH_2_O control and 10.0 + 0.0 treatments, whereas the 10.0 + 5.0 treatment showed the highest mean root length (Figure 2B).

### 2.2. Biochemical Analyses

#### 2.2.1. Chlorophyll Content

**Figure 3:**
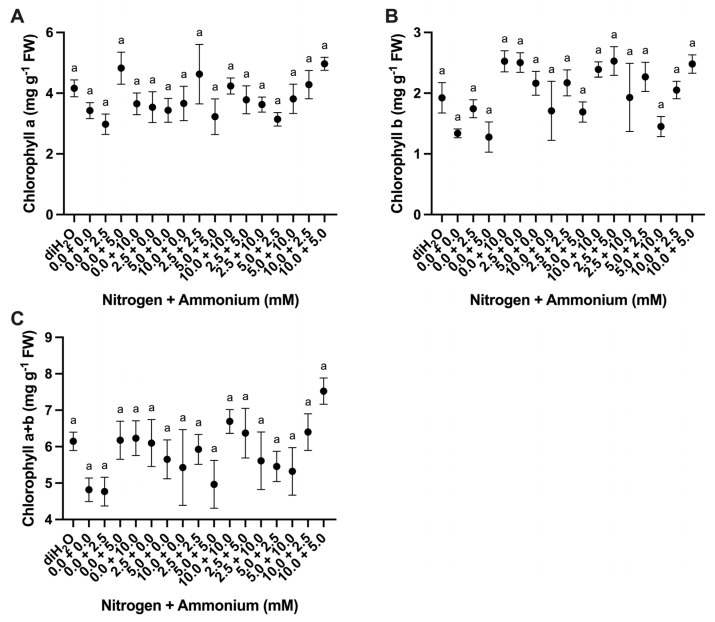
Chlorophyll content changes in cladodes in response to nutrient treatments. (**A**) Chlorophyll a content among treatments (*n* = 6). (**B**) Chlorophyll b content among treatments (*n* = 6). (**C**) Chlorophyll a + b content among treatments (*n* = 6). Treatments consisted of modified Hoagland’s solution with varying amounts of nitrate and ammonium (mM) and a deionized water treatment (diH_2_O) control. Plots show the mean values ± SEM. Letters represent the result of Tukey’s multiple comparisons test (α = 0.05).

Cladodes were evaluated for changes in chlorophyll content as a response to the different nutrient treatments. Chlorophyll a content showed a significant difference among treatments, whereas chlorophyll b content showed an extremely significant difference among analyses (Table 3). However, Tukey’s multiple comparison test of mean values showed no significant differences among the different nutrient treatments for chlorophyll a, chlorophyll b, or chlorophyll a + b (Figure 3A–C). However, the 10.0 + 5.0 treatment consistently showed the highest mean values, whereas the lowest values were observed in the 0.0 + 0.0, 0.0 + 2.5, and 0.0 + 5.0 treatments (Figure 3A–C).

#### 2.2.2. Titratable Acidity

**Figure 4:**
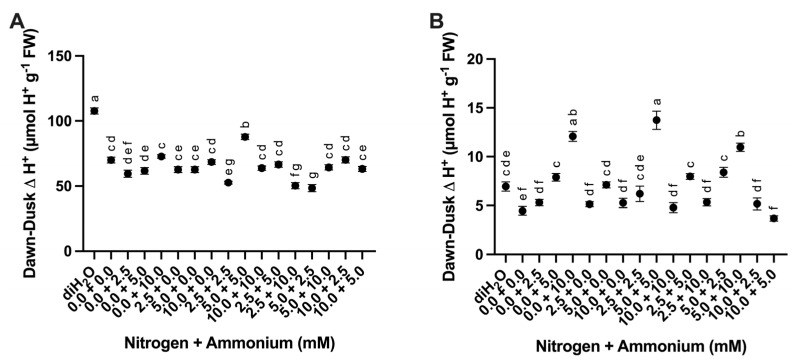
Difference between dawn–dusk titratable acidity changes in cladodes in response to nutrient treatments. (**A**) Titratable acidity to pH = 7.0 (malate equivalents) among treatments (*n* = 6). (**B**) Titratable acidity to pH = 7.0 to 8.4 (citrate equivalents) among treatments (*n* = 6). Treatments consisted of modified Hoagland’s solution with varying amounts of nitrate and ammonium (mM) and a deionized water treatment (diH_2_O) control. Plots show the mean values ± SEM. Letters represent the result of Tukey’s multiple comparisons test (*α* = 0.05).

Cladodes were evaluated for changes in titratable acidity measured at dawn and dusk as a response to the different nutrient treatments. Dawn–dusk H^+^ to pH 7.0 representing malate equivalents showed significant differences among analyses (Table 3) with the highest mean tissue acidity measured in the diH_2_O control treatment (Figure 4A). Dawn–dusk H^+^ from pH 7.0 to pH 8.4 also showed significant differences among treatments (Table 3), with the highest mean tissue acidity evident in the 5.0 + 5.0 treatment (Figure 4B).

#### 2.2.3. Sugar and Starch Content

**Figure 5:**
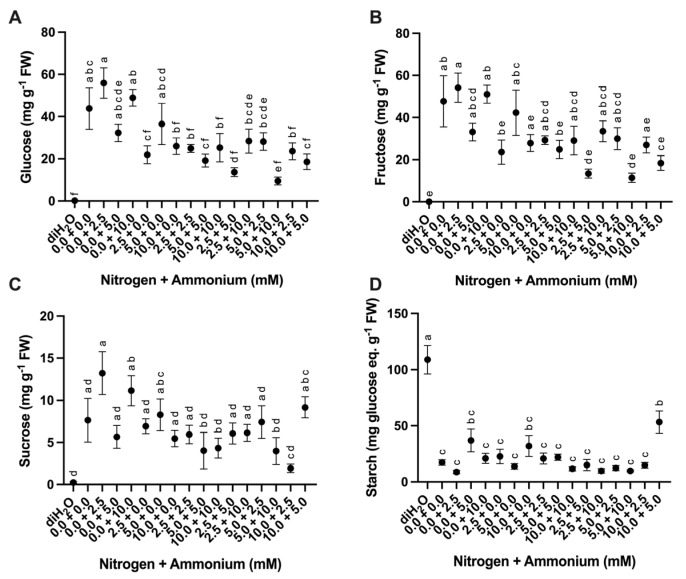
Difference in soluble sugars and starch in cladodes in response to nutrient treatments. (**A**) Glucose content among treatments (*n* = 6). (**B**) Fructose content among treatments (*n* = 6). (**C**) Sucrose content among treatments (*n* = 6). (**D**) Starch content among treatments (*n* = 6). Treatments consisted of modified Hoagland’s solution with varying amounts of nitrate and ammonium (mM) and a deionized water treatment (diH_2_O) control. Plots show the mean values ± SEM. Letters represent the result of Tukey’s multiple comparisons test (α = 0.05).

Cladodes were evaluated for changes in sugar and starch content as a response to the different nutrient treatments. Soluble sugar (glucose, fructose, and sucrose) and starch showed significant differences among treatments (Table 3). Glucose, fructose, and sucrose content was consistently lowest in the diH_2_O control treatments (Figure 5A–C). Across all treatments, mean glucose and fructose contents were consistently higher than mean sucrose content. Furthermore, glucose, fructose, and sucrose contents were highest in the 0.0 + 2.5 treatment (Figure 5A–C). In contrast, starch content was highest in the diH_2_O control treatments, intermediate in the 10.0 + 5.0 treatment, and lower in the remaining treatments (Figure 5D).

#### 2.2.4. Nitrate Reductase Activity

**Figure 6:**
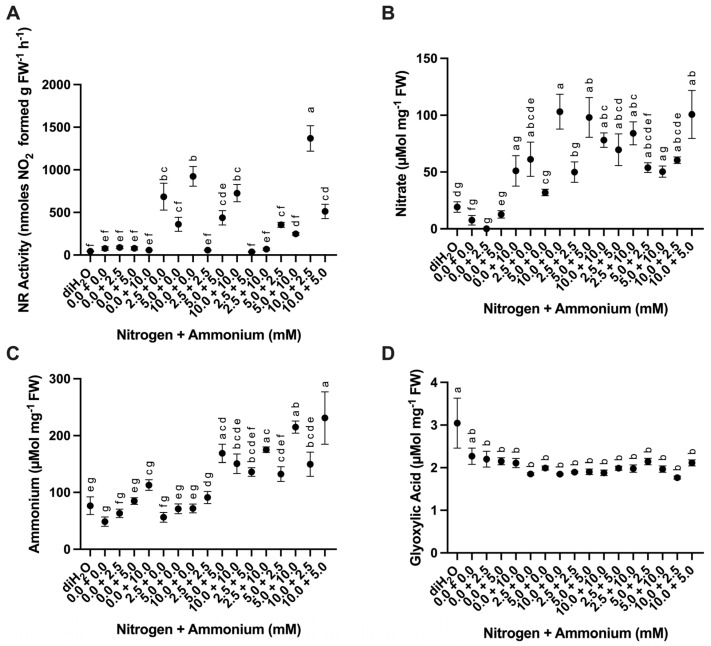
Difference in nitrate reductase (NR) activity in roots and nitrate, ammonium, and glyoxylic acid content in cladodes in response to nutrient treatments. (**A**) Root NR activity among treatments (*n* = 3). (**B**) Nitrate content among treatments (*n* = 6). (**C**) Ammonium content among treatments (*n* = 6). (**D**) Glyoxylic acid content among treatments (*n* = 6). Treatments consisted of modified Hoagland’s solution with varying amounts of nitrate and ammonium (mM) and a deionized water treatment (diH_2_O) control. Plots show the mean values ± SEM. Letters represent the result of Tukey’s multiple comparisons test (α = 0.05).

NR activity measured in the *O. ficus-indica* roots of the plants submitted to different treatments was significantly different (Table 3). The highest NR activity was measured in the 10.0 + 2.5 treatment, whereas the lowest was observed in the 2.5 + 5.0 and diH_2_O treatments (Figure 6A). Roots showed a higher nitrate reductase (NR) activity (mean of 171.0 nmoles NO_2_ g FW^−1^ h^−1^) than cladode chlorenchyma (21 nmoles NO_2_ g FW^−1^ h^−1^) and hydrenchyma (16 nmoles NO_2_ g FW^−1^ h^−1^) tissues. Roots with cortex tissues also showed less NR activity than roots in which the cortex was physically removed before analysis.

#### 2.2.5. Nitrate, Ammonium, and Glyoxylic Acid Content

Cladodes’ nitrate and ammonium content showed significant differences among treatments (Table 3). Mean nitrate content was highest in the 10.0 + 0.0 treatment and lowest in the 0.0 + 2.5 treatment (Figure 6B). The ammonium content was highest in the 10.0 + 5.0 treatment, whereas the 0.0 + 0.0 treatment was the lowest (Figure 6C). Glyoxylic acid content was also significantly different among treatments (Table 3). The diH_2_O control and the 0.0 + 0.0 treatment showed the highest mean glyoxylic acid content (Figure 6D).

#### 2.2.6. Carbon, Nitrogen, and Carbon/Nitrogen Ratios

**Figure 7:**
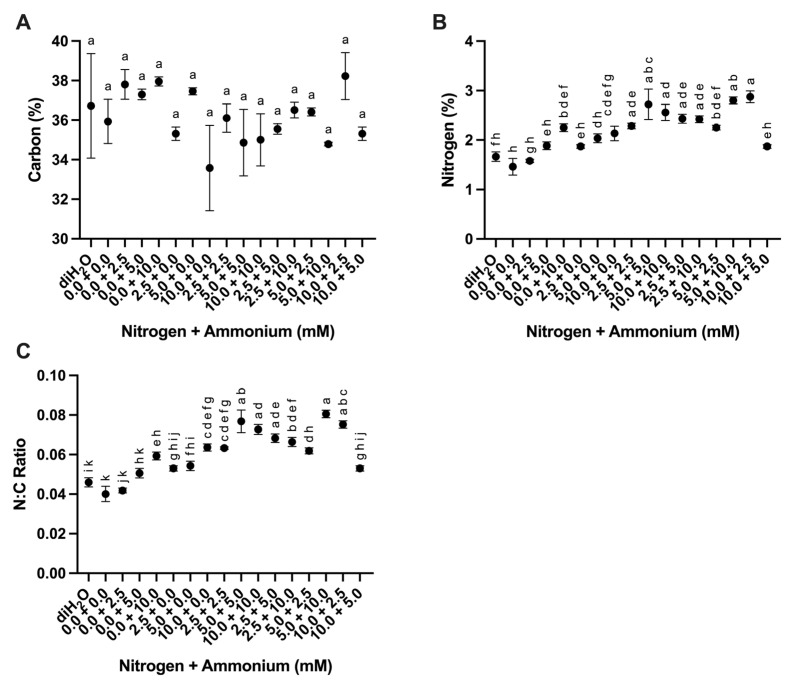
Carbon percentage, nitrogen percentage, and carbon/nitrogen ratios in cladodes in response to nutrient treatments. (**A**) Carbon (%) among treatments (*n* = 3). (**B**) Nitrogen (%) among treatments (*n* = 6). (**C**) Carbon/nitrogen (C:N) ratios among treatments (*n* = 6). Treatments consisted of modified Hoagland’s solution with varying amounts of nitrate and ammonium (mM) and a deionized water treatment (diH_2_O) control. Plots show the mean values ± SEM. Letters represent the result of Tukey’s multiple comparisons test (α = 0.05).

Cladode nitrogen percentage and nitrogen/carbon ratio (N:C) showed significant differences among treatments (Table 3). In contrast, carbon percentage was not significantly different (Table 3). No significant differences in carbon percentage were observed among the treatments (Figure 7A). However, significant differences in nitrogen percentages were evident with nitrogen percentages increasing as greater nitrogen inputs were applied with the highest percent N occurring in the 10.0 + 2.5 treatment (Figure 7B). The N:C ratio generally tracked with the nitrogen inputs with the highest N:C ratio occurring in the 5.0 + 10.0 treatment (Figure 7C).

### 2.3. mRNA Abundance of CAM and N Metabolism-Related Genes

**Figure 8:**
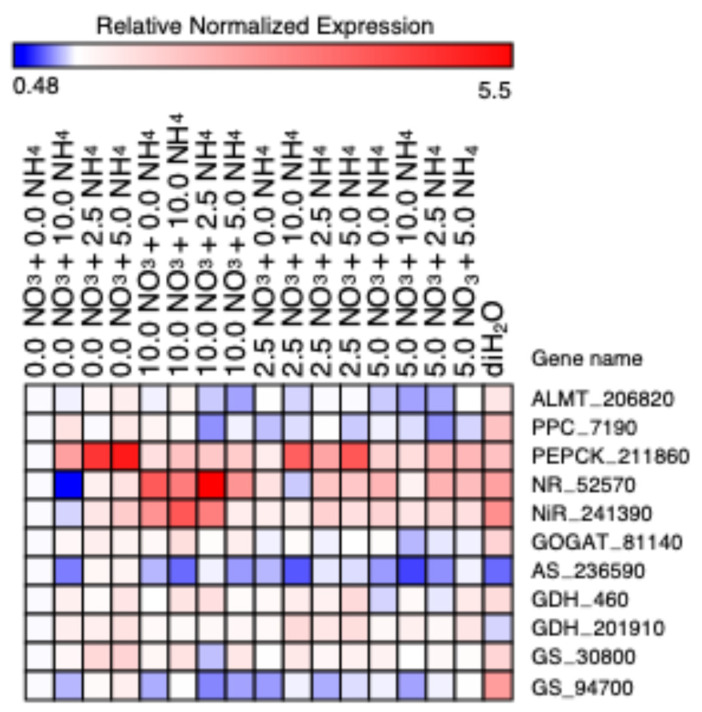
Collective heatmap of CAM-related and N metabolism relative gene expression measured through RT-qPCR analysis among the nitrate and ammonium treatments (mMol). Genes listed: aluminum-activated malate transporter (ALMT_206820), phosphoenolpyruvate carboxylase (PPC_7190), phosphoenolpyruvate carboxykinase (PEPCK_211860), nitrate reductase (NR_52570), nitrite reductase (NiR_241390), glutamate synthase (GOGAT_81140), asparagine synthase (AS_236590), glutamate dehydrogenase (GDH_460, GDH_201910), and glutamine synthetase (GS_30800 and GS_94700). Relative expression of all genes was normalized to the 0 + 0 nitrate and ammonium treatment. The color scale represents actin and ubiquitin normalized log_2_ transformed relative counts where blue indicates low expression and red indicates high expression.

All measured relative expression levels for each gene were significantly different among treatments (Table 4, Figure 8). Of the CAM enzymes surveyed, the steady-state transcript abundance of aluminum-activated malate transporter (ALMT_206820) and phosphosphoenolpyrvate carboxylase (PPC_7190) were the highest in the diH_2_O treatment (Figure 8, Figures S2 and S3). In contrast, the relative steady-state transcript abundance of phosphosphenolpyruvate carboxykinase (PEPCK_211860) was highest in the 0.0 + 2.5 and 0.0 + 5.0 treatments and lowest in the 0.0 + 0.0 treatment (Figure 8 and Figure S4).

For N metabolism genes, the relative steady-state transcript abundance for nitrate reductase (NR_52570) was significantly increased in the 10.0 + 0.0 and 10.0 + 2.5 treatments and was the lowest in the 0.0 + 10.0 treatment (Figure 8 and Figure S5). Nitrite reductase (NiR_241390) steady-state transcript abundance was highest in the 10.0 + 10.0 treatment, and lowest in the 0.0 + 10.0 treatment (Figure 8 and Figure S6). Steady-state transcript abundance of glutamine oxoglutarate aminotransferase (GOGAT_81140) was significantly increased in the 10.0 + 10.0 and diH_2_O treatments, and lowest in the 5.0 + 10.0 treatment (Figure 8 and Figure S7). Asparagine synthase (AS_236590) steady-state transcript abundance was highest in the 0.0 + 5.0 treatment and lowest in the 5.0 + 10.0 treatment (Figure 8 and Figure S8). Glutamate dehydrogenase (GDH_460) steady-state transcript abundance was highest in the 2.5 + 5.0 treatment and diH_2_O treatment and lowest in 5.0 + 0.0 treatment (Figure 8 and Figure S9). Glutamine dehydrogenase (GDH_201910) steady-state transcript abundance was highest in the 2.5 + 10.0 treatment and lowest in the diH_2_O (Figure 8 and Figure S10). Lastly, glutamine synthase (GS_30900) showed no significant difference in steady-state transcript abundance among the treatments (Figure 8 and Figure S11), whereas glutamine synthetase (GS_94700) had about a 3-fold higher steady-state transcript abundance in the diH_2_O treatment than in all other treatments (Figure 8 and Figure S12).

## 3. Discussion

We performed a comprehensive assessment of the nitrate and ammonium preferences of cactus pear cladodes grown under short-term, greenhouse conditions. After one month of acclimating *O. ficus-indica* cladodes to sand culture with diH_2_O and one additional month of varying nitrate and ammonium concentrations in applied nutrient solutions (Table 1), significant differences were observed among treatments for all independent variables measured except for cladode length and width and relative water content (Table 2, Figure 1A,B and Figure S1) and chlorophyll a + b content and percent carbon (Table 3, Figure 3C and Figure 7A). Thus, the one-month acclimation and one-month treatment periods were long enough to elicit significant differences in growth and biochemical parameters, along with changes in gene expression in *O. ficus-indica* (Figure 8 and Figures S2–S12).

The greatest growth stimulation, as measured by the number of new cladodes, occurred with treatments containing a greater amount of nitrate than ammonium (2.5 + 0.0, 5.0 + 2.5, 10.0 + 0.0, 10.0 + 2.5 and 10.0 + 5.0) or with treatments containing higher amounts of nitrate (2.5 + 10, 10 + 0.0) or ammonium (10.0 + 0.0) or a combination of nitrate and ammonium (2.5 + 10.0, 5.0 + 10.0, 2.5 + 2.5, 10.0 + 10.0, 5.0 + 10.0, and 10.0 + 5.0) (Figure 1D). Cladodes treated with only diH_2_O failed to add new cladodes (Figure 1D). Cladode length and width (Figure 1A,B) did not vary greatly among treatments, but cladode thickness did (Figure 1C). Notably, cladode thickness has been shown to correlate strongly with relative water content [22], but relative water content did not vary significantly among treatments within this experiment (Figure S1).

In contrast, although root responses showed more variability, the greatest increase in root number occurred when no nutrients were applied (0.0 + 0.0) or with greater nitrate than ammonium (5.0 + 2.5) (Figure 2A). Similarly, primary root lengths were longer when a greater amount of nitrate than ammonium (10.0 + 5.0) was applied, but results from other combinations were less obvious (Figure 2B). In most plants, including *O. ficus-indica*, limited N in the soil promotes root growth [21,23]. The formation of new biomass might have affected the applied concentrations of nitrate and ammonium due to the mobilization of nutrients between source tissue in mother cladodes and sink tissue in daughter cladodes [24]. Overall, these results suggest that fertilizers designed for *O. ficus-indica* production should have either more nitrate than ammonium or a combination of nitrate and ammonium. These results are also consistent with previous literature reports [21,23].

The observed differences in chlorophyll a, chlorophyll b, and chlorophyll a + b were not significant across the nutrient treatments, with chlorophyll b clearly trending higher in response to a majority of nutrient treatments with the highest values occurring for the nitrate and ammonium (10.0 + 5.0) treatment (Figure 3). In *O. ficus-indica,* chlorophyll content has been shown to increase with increasing amounts of nitrate [25] and to decrease under high light and elevated CO_2_ conditions [26]. Interestingly, diH_2_O control cladodes showed the highest accumulation of organic acids (malate) (Figure 4A), and relative expression of ALMT_206820 (Figure S2) and PPC_7190 genes (Figure S3), which suggests that CAM increased without nutrient provision. Studies in other CAM species have shown that nitrate application can increase CAM activity [20,27]. However, organic acid build-up in the diH_2_O treatment might also be a stress response by the *O. ficus-indica* cladodes [28], rather than an increase in net CO_2_ fixation. The increased accumulation of malate (and other organic acids such as citrate and isocitrate) appears to be a general response to N limitation as observed in rice [29]. Furthermore, these results are consistent with recent evidence that N deficiency is perceived generally as a stress as supported by the enhanced activities of protective antioxidant enzymes and accumulation of sulfur-containing compounds in rice following decreased N supply [30].

In *O. ficus-indica*, fructose, glucose, and sucrose levels make up 35%, 32%, and 33% of the relative sugar content, respectively, found in chlorenchyma, and 44%, 43%, and 13%, respectively, in the parenchyma under well-watered conditions [31]. Homogenized samples (combined chlorenchyma and parenchyma) were analyzed for soluble sugars. We found that mean fructose content was slightly higher than glucose content in all nitrate vs. ammonium treatments (Figure 5A,B) and that sucrose content was lower than both of these monosaccharides (Figure 5C). Interestingly, no measurable soluble sugar content was observed in the diH_2_O control treatment samples (Figure 5A–C). In contrast, the diH_2_O control treatment samples showed the highest starch content (Figure 5D). Rapid starch accumulation under nutrient limitation is a commonly observed response in plants with an associated increase in transcripts and activities of starch biosynthesis enzymes [32]. Thus, under nutrient-limiting conditions, *O. ficus-indica* apparently converts soluble sugars to starch until nutrient availability becomes more favorable, as has been seen in other plant species [33,34,35].

Nitrate reductase activity and nitrate content have been measured in *O. ficus-indica* cladodes and roots under both field and glasshouse conditions [25]. Consistent with their study, we demonstrated increased NR activity in roots (Figure 6A) and nitrate content in cladodes (Figure 6B) when nitrate concentrations were increased. However, NR activity did not always increase when both nitrate and ammonium were present, specifically in the 2.5 + 2.5, 2.5 + 5.0, and 2.5 + 10.0 treatments (Figure 6A). NR activity was observed under higher nitrate concentration when compared to ammonium concentration, which is likely because NO_3_^−^ is the substrate for NR. The highest NR activity was found in new cladodes in contrast to basal cladodes and roots, which showed the least amount of NR activity [25]. This difference is likely because cortical root tissue was removed before conducting NR activity assays, and Nerd and Nobel (1995) used intact roots for measurements [25]. NR in the roots reduces nitrate to nitrite using ferredoxin-reducing power prior to transport to photosynthetic tissues, whereas the nitrate reductase in photosynthetic tissues uses NADH-reducing power [1]. The NR activity measured in this study likely represents the conversion of nitrate to nitrite before being converted into ammonium by nitrate reductase prior to being assimilated into amino acids or transported to the shoots, whereas NR activity in cladodes is likely linked to the conversion of nitrate to nitrite prior to assimilation into amino acids via the GOGAT cycle in plastids [36]. Thus, in *O. ficus-indica,* both root and cladode NR activities increased with an increase in supplied nitrate [25]. However, NR activity and expression are also known to be regulated by light [37]. Nitrate and ammonium content in cladodes were both significantly different across treatments (Figure 6B,C), but understanding these differences is complicated by the fact that ammonium and nitrate are readily interconvertible in both the roots and cladodes [36,38]. Glyoxylic acid content (Figure 6D) was the same across all treatments except for the diH_2_O control supporting the possibility that photorespiration rates in *O. ficus-indica* remained similar regardless of N supply.

Significantly higher uptake of N was observed in *O. ficus-indica* given nitrate vs. ammonium until 20 days after application [21]. Higher above and below ground biomass was also observed when *O. ficus-indica* was provided with nitrate vs. ammonium [21]. Our results complement these former results by demonstrating that the percentage of N appears to be slightly higher in *O. ficus-indica* cladodes when plants were supplied with only nitrate vs. only ammonium in the 2.5 + 0.0 vs. 0.0 + 2.5, and 5.0 + 0.0 vs. 0.0 + 5.0 treatments (Figure 7B). However, no statistically significant differences were observed in the 10.0 + 0.0 vs. 0.0 + 10.0 treatments (Figure 7B). In addition, *O. ficus-indica* cladodes showed a higher percentage of N when supplied with both nitrate and ammonium except in the 10.0 + 5.0 treatment (Figure 7B), which might be a result of nitrate toxicity [39].

In facultative CAM plants, which switch from C_3_ photosynthesis to CAM under unfavorable conditions [40], CAM induction can occur when either high concentrations of an unfavorable N source is present and/or when a favorable source of N is limited [41]. In obligate CAM plants, such as cactaceae species, the proportion of nocturnal CO_2_ uptake by CAM increases when sufficient amounts of N are provided [20]. Indeed, steady-state transcript abundance for PPC_7190 increased in 0.0 + 5.0 and 0.0 + 10.0 ammonium treatments and in the 5.0 + 0.0 and 10.0 + 0.0 nitrate treatments, although this trend was not apparent in the combined nitrate and ammonium treatments (Figure S3). ALMT_206820 steady-state transcript abundance was also higher in 0.0 + 5.0 and 10.0 + 10.0 treatments (Figure S2). An increase in ALMT activity with increases in N might result in an increase in transport of malate into and out of tonoplasts within these treatments, as metabolism, in general, may be increased in this situation [42]. Like PPC_7190, steady-state transcript abundance for PEPCK_211860 increased in response in the 0.0 + 2.5 and 0.0 + 5.0 ammonium treatments, as well as in the combined nitrate and ammonium treatments (2.5 + 5.0 and 2.5 + 10.0) (Figure S4).

NR_52570 and NiR_241390 from cladode tissue showed similar relative expression patterns among the various treatments (Figures S5 and S6), which was likely due to a required coordination of both enzymes for initial nitrate fixation [43]. In addition, the steady-state mRNA abundance of these two enzymes has been shown to increase when N supply is increased, especially in the form of nitrate. In the next step of the pathway, GS fixes ammonium into glutamine both in the cytoplasm through GS1 (GS_30800) and chloroplast with GS2 (GS_94700) [44]. The relative steady-state transcript abundance of GS_30800 and GS_94700 was relatively similar across treatments with the exception that GS_94700 showed increased abundance in the diH_2_O control treatment (Figure S12), whereas GS_30900 relative mRNA expression was similar in all treatments (Figure S11). This observation suggests that chloroplastic GS is upregulated more so than cytosolic GS under nutrient deprivation in *O. ficus-indica*. Likewise, an increase in GOGAT_81140 steady-state transcript abundance was also observed within the diH_2_O control treatment (Figure S7). GOGAT mRNA expression was also significantly higher in the 10.0 + 10.0 treatment (Figure S7), suggesting that GOGAT expression in *O. ficus-indica* was higher when the most N was supplied and when nutrients were limited. The high relative mRNA expression of GOGAT_81140 in the 10.0 + 10.0 treatment was likely due to an increased fixation of glutamine into glutamate with more nitrogen availability, whereas the high GOGAT_81140 relative mRNA expression in the diH_2_O control treatment might be due to glutamate production. Glutamate production has been linked to maintenance of redox homeostasis and ATP production via glycolysis when malate levels are high or NAD-MDH function is lacking [45], as high malate levels (H^+^ equivalent at pH 7.0) were also observed in the diH_2_O treatment (Figure 4A). Asparagine synthetase (AS_236590) steady-state transcript abundance in the cytosol was highest in the ammonium-only (0.0 + 2.5, 0.0 + 5.0) treatments, but lowest in the high N treatments (10.0 + 10.0, 2.5 + 10.0, 5.0 + 10.0) and the diH_2_O control treatment (Figure S8). Under high concentrations of ammonium, GDH converts ammonium into glutamate [46]. The highest steady-state transcript abundance of GDH_460 was measured in the 2.5 + 5.0 treatment (Figure S9), and the highest GDH_201910 steady-state transcript abundance was observed in the 2.5 + 10.0 treatment (Figure S10). GDH_460 showed higher steady-state transcript abundance in the diH_2_O control treatment compared to GDH_20190, and both of these genes showed slightly higher steady-state transcript abundance in the 0.0 + 5.0 treatment than the 0 + 10 treatments (Figures S9 and S10), corroborating previous results [46].

## 4. Materials and Methods

### 4.1. Greenhouse Experimentation and Sample Collection

Prior to planting, 102 mature, approximately one year old, distal, daughter cladodes were collected from 4-year-old *Opuntia-ficus indica* (L.) Mill. plants located in the Valley Road Greenhouse Complex at the University of Nevada, Reno. The original mature plants were grown in three-gallon pots containing a 3:1 ratio of Sunshine MVP soil mix (Sun GroHorticulture, Bellevue, WA, USA) and sand (Sakrete natural play sand, Charlotte, NC, USA) with the cladode placed abscised end down and 5 cm into the soil. Plants were watered once per week during the winter and spring months and twice per week during the summer and fall months. Miracle Gro^®^ fertilizer (Scott’s MiracleGro, Inc., Marysville, OH, USA) and Marathon^®^ 1% Granular insecticide (OHP, Mainland, PA, USA) were applied every six months according to manufacturer’s instructions. Cacti were re-potted on an annual basis. The collected daughter cladodes were allowed to callus for two weeks under greenhouse shaded conditions to prevent infection upon planting. Cladodes were then planted in 11.3 L plastic pots containing a base layer of gravel and the remaining volume with sand (Sakrete natural play sand, Charlotte, NC, USA) sterilized via autoclave set to a 40 min dry cycle at 121 °C and 15 PSI. All plants received 1 L of deionized H_2_O for 1 month prior to applying nutrient treatments to allow for acclimation and to leech any mineral nutrients out of the sand. After acclimation, six cladodes were randomly selected for each nutrient treatment. The position of each potted cladode in the greenhouse was randomized to mitigate any possible differences in microclimate. Under standard greenhouse conditions, the natural light was approximately 1100–1500 µmol m^−2^·s^−1^ and temperature was 28–32 °C day/17–18 °C night.

The experiment was conducted in a cross-factorial design with respect to nitrate and ammonium concentrations (Table 1). Each treatment received 1 L, twice a week, of an assigned modified full Hoagland’s solution with 0.0, 2.5, 5.0, or 10.0 mmol of nitrate and/or ammonium that was adjusted to pH = 5.7–5.8 (Tables S1 and S2). Plants were watered with 1 L of deionized H_2_O (di H_2_O) twice a week as a negative control. After one month of applying treatments, cladodes were collected to measure the parameters detailed below.

### 4.2. Growth Measurements

After 1-month of greenhouse acclimation and before beginning treatments, cladode length, width, new cladode number, root length, and root number were measured. Measurements were taken again one-month following the treatment period. Center cladode thickness was also measured with a digital caliper (IP54 caliper, Baleigh Industrial, Manitowoc, WI, USA) after the treatment period.

### 4.3. Relative Water Content

A 2 cm diameter cork borer was used to collect tissue from each cladode for relative water content. Samples were immediately weighed and submerged in deionized water for 24 h and weighed again to determine the turgid weight. Lastly, samples were dried for 72 h in a lyophilizer (7755030, Labconco, Inc., Kansas City, MO, USA) and weighed to determine dry weight. The relative water content was calculated as:

$$RWC\;\left(\%\right)=\left(\frac{w-d}{t}-d\right)*100\left(\%\right)$$

where *w* is fresh weight, *t* is turgid weight, and *d* is dry weight all in grams.

### 4.4. Chlorophyll Content

Chlorophyll content was determined using a protocol modified from [47]. Three hundred mg of frozen and ground tissue was placed into 15 mL Falcon tubes (430791, Corning, Corning, NY, USA) and mixed with 5 mL of 80% acetone in the dark. Samples were then centrifuged at 3000× *g* at 4 °C for 15 min and preserving the supernatant. The supernatant was loaded into disposable cuvettes and the absorbance at 663 nm for chlorophyll a (Ca) and 645 nm for chlorophyll b (Cb) was measured using a Nanodrop 2000 spectrophotometer (Thermo Fisher Scientific, Waltham, MA, USA). The chlorophyll content was calculated as:

$$Ca\;\left(mg/g\;sample\right)=\left(12.7*A-2.69*B\right)*\frac{v}{1000}*w$$

$$Cb\;(mg/g\;sample)=\left(22.9*B-4.86*A\right)*\frac{v}{1000}*w$$

$$Ca+b\;(mg/g\;sample)=\left(8.02*A+20.20*B\right)*\frac{v}{1000}*w$$

where *A* is absorbance at 663 nm, *B* is absorbance at 645 nm, *v* is volume of extract in ml, and *w* is weight of the sample in g.

### 4.5. Titratable Acidity

To determine the nocturnal acid stored overnight within treatments, between 0.844 and 2.886 g fresh weight material was collected with a 2 cm diameter cork borer at dawn and dusk from each cladode and flash frozen in liquid nitrogen. The titratable acidity was determined using a modified protocol [48]. Collected tissue was ground in a mortar and pestle containing liquid nitrogen. An amount of 0.5 g of ground freeze-dried tissue from each sample was placed in pre-chilled 15 mL conical tubes. Ten mL of 50% methanol was added to each sample and the top volume point marked with a marker. A small hole was made in the cap of each tube, and samples were boiled in an 80 °C water bath for 10 min. After boiling, samples were filled to the marked level with diH_2_O and centrifuged at 3000× *g* for 10 min. The supernatant was decanted into 50 mL beakers and titrated to pH 7.0 for malate equivalence, and pH 8.4 for citrate equivalence with 10 mM KOH. The H^+^ equivalent at 7.0 and 8.4 was calculated by:

$$H^{+}\;equivalent\left(µmol\;H^{+}/gFW\right)=w\left(\frac{0.01}{v}\right)*1000$$

where *w* is the fresh weight of the sample in grams, *v* is the volume of 10 mM KOH added in mL.

For each sample, the total nocturnal concentration of malate was calculated by subtracting the dusk sample H^+^ equivalent from the dawn sample H^+^ equivalent. The total nocturnal concentration of citrate was calculated by subtracting the dusk sample H^+^ equivalent from the dawn sample H^+^ equivalent.

### 4.6. Starch Content and Soluble Sugars

The soluble sugars, glucose, fructose, and sucrose and non-soluble starch contents were analyzed exactly as specified in [49]. Briefly, 10 mg of frozen, ground tissue harvested at noon was used for methanol and chloroform phase separation with the top phase containing soluble sugars and the lower phase containing starch. The top phase containing soluble sugars and the lower phase containing starch were separated for independent analysis. The soluble sugar fraction was analyzed by conducting sequential enzyme assays that measure the production of NADH at 340 nm in a SpectraMax M5 multi-mode microplate reader (Molecular Devices, LLC, San Jose, CA, USA). after the addition of glucose-6-phosphate dehydrogenase (10165875001, Sigma-Aldrich, St. Louis, MO, USA) for glucose content, hexokinase (11426362001, Sigma-Aldrich, St. Louis, MO, USA), and phosphogluco-isomerase (10128139001, Sigma-Aldrich, St. Louis, MO, USA) for fructose content, and β-fructosidase (14504, Sigma-Aldrich, St. Louis, MO, USA) for sucrose content, respectively. The lower-starch-containing phase was hydrolyzed into glucose monomers by autoclaving followed by the application of an amylglucosidase (11202332001, Sigma-Aldrich, St. Louis, MO, USA) treatment. The freed glucose monomers were then determined by measuring the production of NADH at 340 nm after the addition of glucose-6-phosphate dehydrogenase (10165875001, Sigma-Aldrich, St. Louis, MO, USA) as in the soluble sugar assay.

### 4.7. Nitrate Reductase Activity

For nitrate reductase (NR) activity assays, roots were collected from each cladode, and the cortex was removed by hand before recording fresh weight. Prior to experimentation, a phosphate buffer was made by combining 500 mL of 0.1 M KH_2_PO_4_ with 400 mL of 0.1 M NaOH until pH = 7.5 was achieved using 0.1 M NaOH. An amount of 1 L of an incubation buffer was made by adding 970 mL of the phosphate buffer with 30 mL of *n*-propanol and 100 mM KNO_3_. The incubation buffer was heated in a water bath for 20 min at 30 °C and then placed in a Sonic Dismembrator (F60, Fisher Scientific, San Diego, CA, USA) and vacuum pump for 15 min to eliminate O_2_ from the solution. The incubation solution void of O_2_ was then kept in a water bath at 30 °C until fresh tissue was collected.

Approximately 0.5 g of *O. ficus-indica* cortex-free root tissue was placed into 15 mL glass tubes. Six ml of the O_2_-free incubation buffer was added to each sample, and all samples were placed into a vacuum chamber for two rounds of 1 min each to promote infiltration of tissues with the incubation solution. All samples were kept in the dark or under foil for the remainder of the experiment to prevent nitrate degradation by light. An amount of 1 mL of incubation buffer from each sample was then pipetted into 2 mL microtubes to represent time point 0 (T0). T0 tubes were incubated at room temperature for one hour. The remaining samples were incubated for 1 h in a 30 °C water bath, and 1 mL was transferred to a second set of tubes to represent the 60 min time point (T60). One mL of O_2_-free incubation buffer was added in a separate microtube as a blank for spectrophotometer readings and final calculation. In each microtube, i.e., T0, T60, and blank, 30 µL of 1% sulfanilamide in 3 M HCl was added and vortexed. Then, 300 µL of 0.02% of N-(1-Naphthyl) ethylenediamine dihydrochloride in Nanopure water was added and vortexed, and samples were allowed to incubate for 30 min at room temperature. Lastly, samples were loaded into quartz cuvettes and measured at 540 nm in a Nanodrop 2000 spectrophotometer (Thermo Fisher Scientific, Waltham, MA, USA) using the appropriate buffer blank. A 345 mg NaNO_2_ in 500 mL water solution was diluted to make 0, 1, 2, 4, 8, and 16 µM NO_2_^−^/L standard solutions.

The reaction rate of nitrate reductase in solution was calculated by first converting the T0 and T60 measurements to a µM concentration using the equation of the best fit line of the standard absorbance readings. This calculated concentration was normalized by dividing it by the initial sample weight. Lastly, subtracting the normalized T0 concentration from the T60 concentration gave the µM of NO_2_ produced per gram of fresh weight of sample per hour by NR.

### 4.8. Nitrate Content

Nitrate content was determined using a modified protocol [50]. Briefly, 20 mg of ground, freeze-dried tissue was resuspended in deionized water and incubated at 45 °C for 1 h. Samples were then mixed and centrifuged at 5000× *g* for 15 min. An amount of 0.2 mL of supernatant was placed into a 50 mL flask with 5% salicylic acid in concentrated H_2_SO_4_ for 20 min at room temperature. Nineteen mL of 2 N NaOH was added to each sample to adjust pH ≥ 12. Flasks were gently vortexed for 5 min, and 100 µL of each sample was loaded into a 96-well clear polycarbonate, flatbottom microliter plate (#3364, Corning, Corning, NY, USA). The absorbance was measured at 410 nm using a SpectraMax M5 multi-mode microplate reader (Molecular Devices, LLC, San Jose, CA, USA). Samples were compared to a set of eight standards containing between 0 and 60 mg of NO_3_^−^ using a KNO_3_^−^ standard solution and normalized by sample dry weight.

### 4.9. Ammonium and Glyoxylic Acid Content

Ammonium and glyoxylic acid content were quantified following [51]. An amount of 50 mg of homogenized freeze-dried tissue of each cladode was mixed with 1 mL of 100 mM HCl and 500 µL of chloroform in 2 mL test tubes. Samples were centrifuged at 12,000× *g* for 5 min at 8 °C. The aqueous phase was transferred to a new set of test tubes containing 50 mg of acid-washed activated charcoal, gently swirled and centrifuged again at 20,000× *g* for 5 min at 8 °C. An amount of 200 µL of the charcoal-washed supernatant was used in the glyoxylate assay, and 200 µL was used in the ammonium assay.

The glyoxylate samples were combined with 20 µL of a 1% (*v*/*v*) solution of phenylhydrazine in 100 mM HCl and incubated in a 95 °C water bath for 2 min and immediately cooled on ice for 6 min. An amount of 100 µL of concentrated HCl was added to each sample. An amount of 225 µL of each sample was loaded into a 96-well clear flatbottom microliter plate. Absorbance was measured at 520 nm at exactly 4, 5, and 6 min after the addition of 25 µL of 1.6% K_3_Fe(CN)_6_ solution using a SpectraMax M5 multi-mode microplate reader (Molecular Devices, LLC, San Jose, CA, USA).

Ammonium samples (200 µL) were diluted 1:1 with 100 mM HCl. An amount of 20 µL of this solution was mixed with 100 µL of 1% (*w*/*v*) phenol, 0.005% (*w*/*v*) sodium nitroprusside solution in water, 100 µL of 1% (*v*/*v*) sodium hypochlorite and 0.5% (*w*/*v*) sodium hydroxide. All samples were then incubated at 37 °C for 30 min, and the absorbance at 520 nm was measured in a SpectraMax M5 multi-mode microplate reader (Molecular Devices, LLC, San Jose, CA, USA). Concentrations were calculated with the equation of a linear curve with 12 ammonium standards between 0 and 20 mM concentrations of ammonium sulfate.

### 4.10. Carbon and Nitrogen Content

Total carbon and nitrogen content was determined by loading approximately 50 mg of ground freeze-dried tissue from each cladode into clay crucibles (2203-828, Leco, St. Joseph, MI, USA) for elemental analysis with a Leco 928 combustion analyzer (Leco, St. Joseph, MI, USA). Results were normalized on a weight basis and presented as the ratio of unit N per unit C (N:C Ratio).

### 4.11. RT-qPCR of CAM- and Nitrogen-Related Genes

To measure the expression of CAM- and nitrogen-metabolism-related genes across treatments, plant tissue was collected at noon with a 2 cm diameter cork borer from each cladode and then immediately frozen in liquid nitrogen and ground to a fine powder using a mortar and pestle. Hundred mg of ground frozen tissue was used for RNA extraction using a modified Qiagen RNeasy Plant Mini Kit (Cat. No. 79254, Qiagen Inc., Redwood City, CA, USA) protocol that included the addition of Fruit-Mate (Cat. No. 9192, Takara Bio Inc., Kusatsu, Shiga, Japan), a proprietary non-ionic polymer that binds to polysaccharides and polyphenols, and DNase digestion. The addition of Fruit-Mate was necessary to perform RNA extractions on *O. ficus-indica* due to the naturally occurring high pectin content [52]. RNeasy kit protocol was followed exactly as specified by the manufacturer with the addition of 1 mL of Fruit-Mate to the samples in step 2 and on-column DNase digestion using the RNase-free DNase kit as specified by Qiagen. The RNA concentration was measured with a Nanodrop 2000 spectrophotometer (Thermo Fisher Scientific, Waltham, MA, USA). Potential RNA degradation during the extraction was checked by electrophoretic separation on a 1% agarose gel with Qiagen RNA sample loading dye (Cat. No. 74904, Qiagen Inc., Redwood City, CA, USA)). cDNA of the extracted RNA transcripts was generated following iScript™ Reverse Transcription Supermix for RT-qPCR protocol (Cat. No. 1708840, Bio-Rad Laboratories, Hercules, CA, USA).

For RT-qPCR analysis, primers were designed for *O. ficus-indica* genes related to CAM and nitrogen metabolism shown in Table S3. Real-time quantification was performed following the SsoAdvanced Universal SYBR Green Supermix (Cat. No. 172-5271, Bio-Rad Laboratories, Hercules, CA, USA) protocol. The relative amounts of cDNA in each sample were determined on the basis of the threshold cycle (Ct) for each PCR product and normalized to both UBQ10 (Op_ fin19) and ACTIN7 (Op_ fin88560) Ct values [53,54]. Predicted localization of the final product of each gene was estimated by first translating the cDNA sequence to protein sequence using the Expasy translate tool (https://web.expasy.org/translate/, accessed on 17 September 2024). Then, the resulting protein sequence was analyzed using LOCALIZER software (http://localizer.csiro.au/, accessed on 16 July 2019) to generate a subcellular localization prediction [55].

### 4.12. Statistical Analysis

All raw data input and calculations described above were performed in using GraphPad Prism 10 software. Analysis of variance for one factor one-way ANOVA and Tukey’s multiple comparisons test (α = 0.05) were performed, and mean data were plotted with standard error of the mean (±SEM).

## 5. Conclusions

*O. ficus-indica* serves as an agriculturally important CAM crop species with many uses. To maximize biomass production for food, feed, and biofuel production, the optimal fertilization requirements need to be better understood. Using a sand culture system and a cross-factorial study design of nutrient solutions containing differing concentrations of nitrate and/or ammonium, our results suggest that *O. ficus-indica* prefers fertilization with either more nitrate than ammonium or various concentrations of nitrate and ammonium as assessed by both cladode and root growth and chlorophyll content. Such information is key to maximizing *O. ficus-indica* production under field conditions. Nutrient deprivation resulted in a stimulation in root growth and a depletion of soluble sugars with a corresponding increase in starch accumulation, which are common responses to nutrient starvation in plants. Nutrient-deprived plants also showed increased accumulation of organic acids (malate + citrate) and steady-state transcript abundance increases for several CAM-related and N metabolism genes, suggesting that CAM or malate content increased as a stress response to lack of nutrient availability or an unfavorable N source similar to what has been observed in other CAM species.

## Figures and Tables

**Table 1:** The cross-factorial design to test the response of *Opuntia ficus-indica* to understand possible synergistic effects with differences in NO_3_^−^ and NH_4_^+^ availability. An additional treatment comprising only receiving deionized water was included (diH_2_O).

Treatments	0.0 mM NO_3_^−^	2.5 mM NO_3_^−^	5.0 mM NO_3_^−^	10.0 mM NO_3_^−^
0.0 mM NH_4_^+^	0.0 mM NH_4_^+^ 0.0 mM NO_3_^−^	0.0 mM NH_4_^+^ 2.5 mM NO_3_^−^	0.0 mM NH_4_^+^ 5.0 mM NO_3_^−^	0.0 mM NH_4_^+^ 10.0 mM NO_3_^−^
2.5 mM NH_4_^+^	2.5 mM NH_4_^+^ 0.0 mM NO_3_^−^	2.5 mM NH_4_^+^ 2.5 mM NO_3_^−^	2.5 mM NH_4_^+^ 5.0 mM NO_3_^−^	2.5 mM NH_4_^+^ 10.0 mM NO_3_^−^
5.0 mM NH_4_^+^	5.0 mM NH_4_^+^ 0.0 mM NO_3_^−^	5.0 mM NH_4_^+^ 2.5 mM NO_3_^−^	5.0 mM NH_4_^+^ 5.0 mM NO_3_^−^	5.0 mM NH_4_^+^ 10.0 mM NO_3_^−^
10.0 mM NH_4_^+^	10.0 mM NH_4_^+^ 0.0 mM NO_3_^−^	10.0 mM NH_4_^+^ 2.5 mM NO_3_^−^	10.0 mM NH_4_^+^ 5.0 mM NO_3_^−^	10.0 mM NH_4_^+^ 10.0 mM NO_3_^−^

**Table 2:** Ordinary one-way analysis (ANOVA) results of growth and relative water content. Significance codes: highly significant *p* < 0.001 ‘**’, significant *p* < 0.01 ‘*’, and not significant *p* > 0.05 ‘NS’.

Independent Variable	Number of Replicates (*n*) Within Treatment	Degrees of Freedom (DF)	F-Value	*p*-Value	Significance Code
Pad length	3	16	1.569	0.1260	NS
Pad width	3	16	2.074	0.0326	*
Pad thickness	6	16	2.678	0.001	**
New cladode #	3	16	2.689	0.0063	**
Primary root length	3	16	2.544	0.0091	**
Root length	3	16	2.097	0.0306	*
Relative water content	6	16	1.533	0.1070	NS

**Table 3:** Ordinary one-way analysis (ANOVA) results of biochemical analyses with six samples (*n* = 6) in each treatment and 17 treatments (DF = 16) in all. Significance codes: extremely significant *p* < 0.0001 ‘***’, significant *p* < 0.01 ‘*’, and not significant *p* > 0.05 ‘NS’.

Independent Variable	F-Value	*p*-Value	Significance Code
Chlorophyll a + b	1.674	0.0676	NS
Chlorophyll a	1.909	0.0303	*
Chlorophyll b	2.909	0.0007	***
Titratable acidity (pH 7)	42.61	<0.0001	***
Titratable acidity (pH 7–8.4)	32.67	<0.0001	***
Starch	16.68	<0.0001	***
Glucose	7.189	<0.0001	***
Fructose	6.279	<0.0001	***
Sucrose	4.171	<0.0001	***
NR Activity	26.22	<0.0001	***
Nitrate	9.492	<0.0001	***
Ammonium	12.38	<0.0001	***
Glyoxylic acid	3.004	0.0001	***
N:C Ratio	24.6	<0.0001	***
Percent C	1.39	0.1634	NS
Percent N	12.65	<0.0001	***

**Table 4:** Ordinary one-way analysis (ANOVA) results of relative gene expression results with 6 samples (*n* = 6) in each treatment and 17 treatments (DF = 16) in all. Significance codes: extremely significant *p* < 0.0001 ‘***’, significant *p* < 0.01 ‘*’.

Gene Name	Enzyme Name	F-Value	*p*-Value	Significance Code
ALMT_206820	Aluminum-activated malate transporter	3.319	0.0001	***
PPC_7190	Phosphoenolpyruvate carboxylase	6.963	<0.0001	***
PEPCK_211860	Phosphoenolpyruvate carboxykinase	3.460	<0.0001	***
NR_52570	Nitrate reductase	10.290	<0.0001	***
NiR_241390	Nitrite reductase	8.182	<0.0001	***
GOGAT_81140	Glutamate synthase	4.176	<0.0001	***
AS_236590	Asparagine synthase	3.885	<0.0001	***
GDH_460	Glutamate dehydrogenase	3.773	<0.0001	***
GDH_201910	Glutamate dehydrogenase	4.068	<0.0001	***
GS_30800	Glutamine synthetase	1.950	0.0263	*
GS_94700	Glutamine synthetase	13.490	<0.0001	***

## Data Availability

The original contributions presented in the study are included in the article/Supplementary Material; further inquiries can be directed to the corresponding author/s.

## Supplementary Materials

The following supporting information can be downloaded at: https://www.mdpi.com/article/10.3390/plants13243489/s1. Figure S1: Percentage of cladode relative water content (RWC) among treatments. Treatments consisted of modified Hoagland’s solution with varying amounts of nitrate and ammonium (mM) and a deionized water treatment (diH_2_O) control. Plots show the mean values with error bars indicating ± standard error of the mean (SEM) (*n* = 3). Letters represent the result of Tukey’s multiple comparisons test (α = 0.05). Figure S2: Relative expression of aluminum-activated malate transporter (ALMT_206820) among nitrate and ammonium treatments (mM). Relative expression of ALMT_206820 in all treatments was normalized to average ALMT_206820 expression in the 0 + 0 nitrate and ammonium treatment. All values represent the average actin and ubiquitin normalized log_2_ transformed counts. Treatments consisted of modified Hoagland’s solution with varying amounts of nitrate and ammonium (mM) and a deionized water treatment (diH_2_O) control. Plots show the mean values with error bars indicating ± SEM (*n* = 6). Letters represent the result of Tukey’s multiple comparisons test (a = 0.05). Figure S3: Relative expression of phosphoenolpyruvate carboxylase (PPC_7190) among nitrate and ammonium treatments (mM). Relative expression of PPC_7190 in all treatments was normalized to average PPC_7190 expression in the 0 + 0 nitrate and ammonium treatment. All values represent the average actin and ubiquitin normalized log_2_ transformed counts. Treatments consisted of modified Hoagland’s solution with varying amounts of nitrate and ammonium (mM) and a deionized water treatment (diH_2_O) control. Plots show the mean values with error bars indicating ± SEM (*n* = 6). Letters represent the result of Tukey’s multiple comparisons test (a = 0.05). Figure S4: Relative expression of phosphoenolpyruvate carboxylase kinase (PEPCK_211860) among nitrate and ammonium treatments (mM). Relative expression of PEPCK_211860 in all treatments was normalized to average PEPCK_211860 expression in the 0 + 0 nitrate and ammonium treatment. All values represent the average actin and ubiquitin normalized log_2_ transformed counts. Treatments consisted of modified Hoagland’s solution with varying amounts of nitrate and ammonium (mM) and a deionized water treatment (diH_2_O) control. Plots show the mean values with error bars indicating ± SEM (*n* = 6). Letters represent the result of Tukey’s multiple comparisons test (a = 0.05). Figure S5: Relative expression of nitrate reductase (NR_52570) among nitrate and ammonium treatments (mM). Relative expression of NR_52570 in all treatments was normalized to average NR_52570 expression in the 0 + 0 nitrate and ammonium treatment. All values represent the average actin and ubiquitin normalized log_2_ transformed counts. Treatments consisted of modified Hoagland’s solution with varying amounts of nitrate and ammonium (mM) and a deionized water treatment (diH_2_O) control. Plots show the mean values with error bars indicating ± SEM (*n* = 6). Letters represent the result of Tukey’s multiple comparisons test (a = 0.05). Figure S6: Relative expression of nitrite reductase (NiR_241390) among nitrate and ammonium treatments (mM). Relative expression of NiR_241390 in all treatments was normalized to mean NiR_241390 expression in the 0 + 0 nitrate and ammonium treatment. All values represent the average actin and ubiquitin normalized log_2_ transformed counts. Treatments consisted of modified Hoagland’s solution with varying amounts of nitrate and ammonium (mM) and a deionized water treatment (diH_2_O) control. Plots show the mean values with error bars indicating ± SEM (*n* = 6). Letters represent the result of Tukey’s multiple comparisons test (a = 0.05). Figure S7: Relative expression of glutamine oxoglutarate aminotransferase (GOGAT_81140) among nitrate and ammonium treatments (mM). Relative expression of GOGAT_81140 in all treatments was normalized to average GOGAT_81140 expression in the 0 + 0 nitrate and ammonium treatment. All values represent the average actin and ubiquitin normalized log_2_ transformed counts. Treatments consisted of modified Hoagland’s solution with varying amounts of nitrate and ammonium (mM) and a deionized water treatment (diH_2_O) control. Plots show the mean values with error bars indicating ± SEM (*n* = 6). Letters represent the result of Tukey’s multiple comparisons test (a = 0.05). Figure S8: Relative expression of asparagine synthase (AS_236590) among nitrate and ammonium treatments (mM). Relative expression of AS_236590 in all treatments was normalized to mean AS_236590 expression in the 0 + 0 nitrate and ammonium treatment. All values represent the average actin and ubiquitin normalized log_2_ transformed counts. Treatments consisted of modified Hoagland’s solution with varying amounts of nitrate and ammonium (mM) and a deionized water treatment (diH_2_O) control. Plots show the mean values with error bars indicating ± SEM (*n* = 6). Letters represent the result of Tukey’s multiple comparisons test (a = 0.05). Figure S9: Relative expression of glutamate dehydrogenase (GDH_460) among nitrate and ammonium treatments (mM). Relative expression of GDH_460 in all treatments was normalized to average GDH_460 expression in the 0 + 0 nitrate and ammonium treatment. All values represent the average actin and ubiquitin normalized log_2_ transformed counts. Treatments consisted of modified Hoagland’s solution with varying amounts of nitrate and ammonium (mM) and a deionized water treatment (diH_2_O) control. Plots show the mean values with error bars indicating ± SEM (*n* = 6). Letters represent the result of Tukey’s multiple comparisons test (a = 0.05). Figure S10: Relative expression of glutamine dehydrogenase (GDH_201910) among nitrate and ammonium treatments (mM). Relative expression of GDH_201910 in all treatments was normalized to mean GDH_201910 expression in the 0 + 0 nitrate and ammonium treatment. All values represent the average actin and ubiquitin normalized log_2_ transformed counts. Treatments consisted of modified Hoagland’s solution with varying amounts of nitrate and ammonium (mM) and a deionized water treatment (diH_2_O) control. Plots show the mean values with error bars indicating ± SEM (*n* = 6). Letters represent the result of Tukey’s multiple comparisons test (a = 0.05). Figure S11: Relative expression of glutamine synthase (GS_30800) among nitrate and ammonium treatments (mM). Relative expression of GS_30800 in all treatments was normalized to mean GS_30800 expression in the 0 + 0 nitrate and ammonium treatment. All values represent the average actin and ubiquitin normalized log_2_ transformed counts. Treatments consisted of modified Hoagland’s solution with varying amounts of nitrate and ammonium (mM) and a deionized water treatment (diH_2_O) control. Plots show the mean values with error bars indicating ± SEM (*n* = 6). Letters represent the result of Tukey’s multiple comparisons test (a = 0.05). Figure S12: Relative expression of glutamine synthetase (GS_94700) among nitrate and ammonium treatments (mM). Relative expression of GS_94700 in all treatments was normalized to mean GS_94700 expression in the 0 + 0 nitrate and ammonium treatment. All values represent the average actin and ubiquitin normalized log_2_ transformed counts. Treatments consisted of modified Hoagland’s solution with varying amounts of nitrate and ammonium (mM) and a deionized water treatment (diH_2_O) control. Plots show the mean values with error bars indicating ± SEM (*n* = 6). Letters represent the result of Tukey’s multiple comparisons test (a = 0.05). Figure S13: Reference gene sequences use for gene expression studies. Forward and reverse primers used are indicated by yellow and green highlighting, respectively (see Table S3). Table S1: Recipes for nutrient working solutions that were stored in the dark at 4 °C and combined to make each treatment nutrient mix prior to application. Table S2: Amount of working solutions combined to make each treatment in ml. The resulting nutrient solution was brought to a final volume of 1.5 L with deionized water. Two-fifty mL of the final solution was added to each of six individual *O. ficus-indica* plants in each treatment twice a week for one month. Table S3: List of primers designed for RT-qPCR analysis with predicted subcellular localizations.
