# Supplementary material for: The Nitrogen Preference of Cactus Pear (Opuntia ficus-indica): A Sand Culture Snapshot
Source: Plants (Basel). 2024 Dec 13;13(24):3489. doi: 10.3390/plants13243489 (PMC11728482; doi:10.3390/plants13243489)
Supplement: Supplementary file 1 [file plants-13-03489-s001.zip › plants-3239151-supplementary.pdf]

Supplementary Materials:

Supplemental Figures

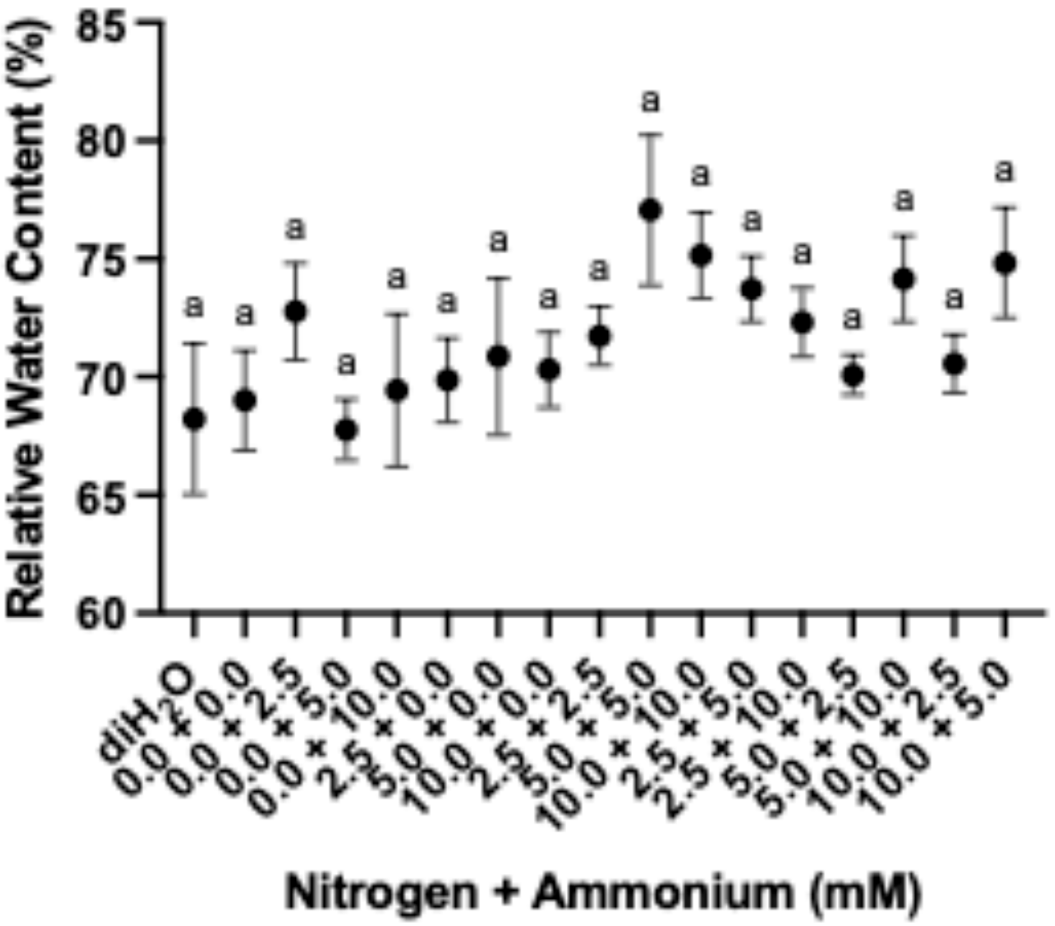

Figure S1: Percentage of cladode relative water content (RWC) among treatments. Treatments consisted of modified Hoagland's solution with varying amounts of nitrate and ammonium (mM) and a deionized water treatment (diH_2O) control. Plots show the mean values with error bars indicating ± standard error of the mean (SEM) (n = 3). Letters represent the result of Tukey's multiple comparisons test (α = 0.05).

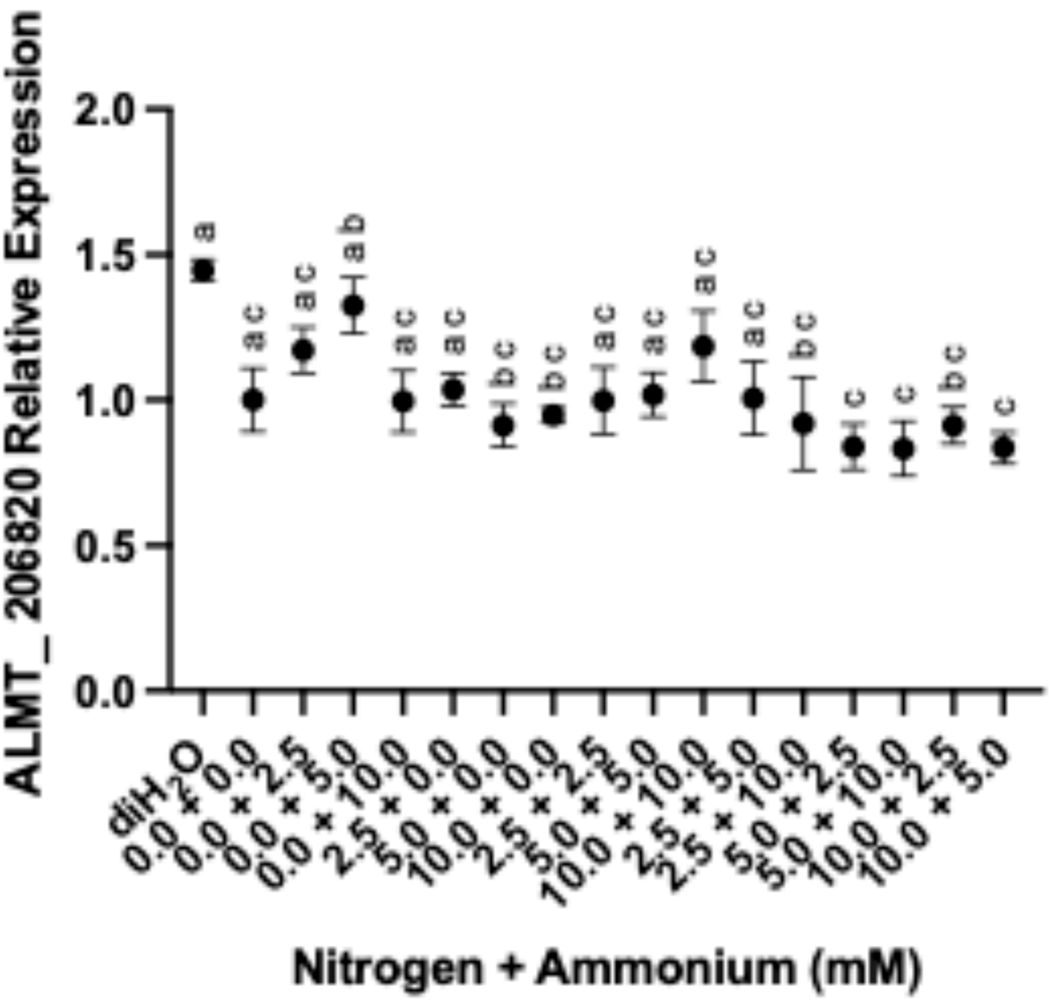

Figure S2: Relative expression of aluminum-activated malate transporter (ALMT_206820) among nitrate and ammonium treatments (mM). Relative expression of ALMT_206820 in all treatments was normalized to average ALMT_206820 expression in the 0+0 nitrate and ammonium treatment. All values represent the average actin and ubiquitin FPKM normalized log_2 transformed counts. Treatments consisted of modified Hoagland's solution with varying amounts of nitrate and ammonium (mM) and a deionized water treatment (diH_2O) control. Plots show the mean values with error bars indicating ± SEM (n = 6). Letters represent the result of Tukey's multiple comparisons test (α = 0.05).

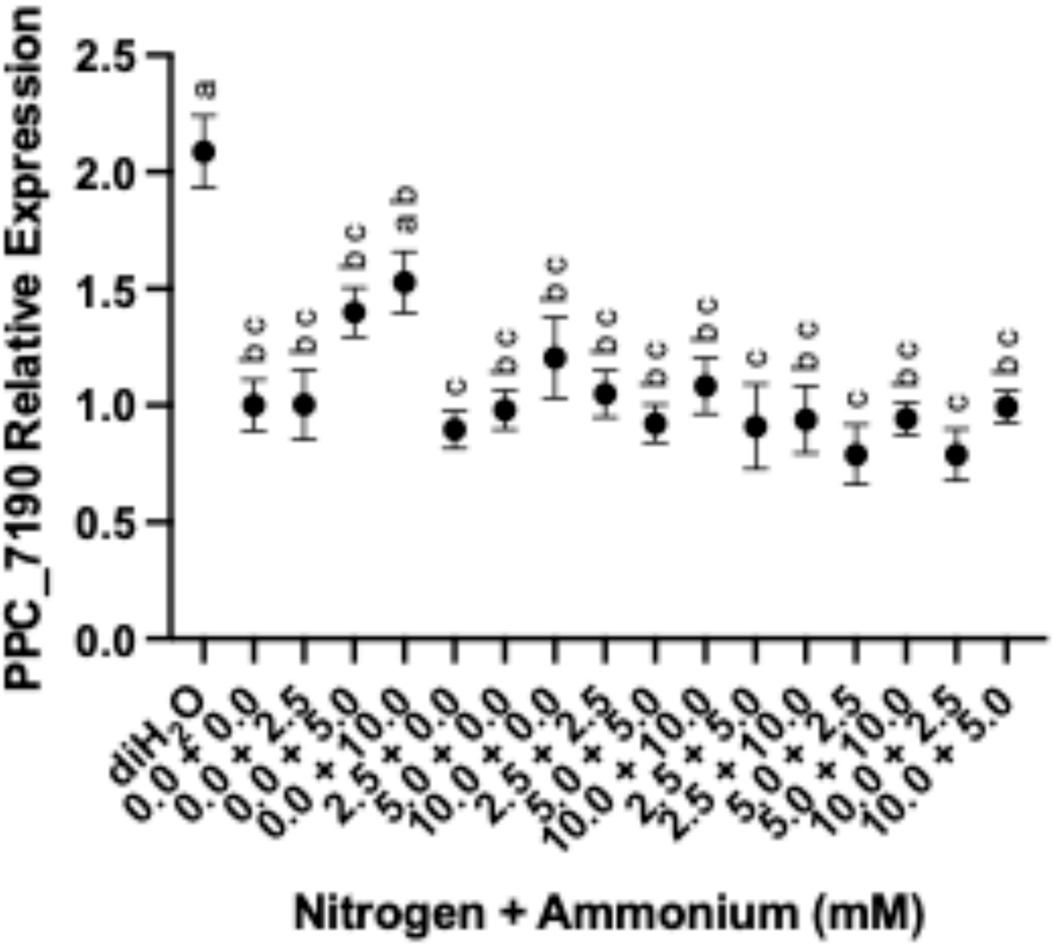

Figure S3: Relative expression of phospho*enol*pyruvate carboxylase (PPC_7190) among nitrate and ammonium treatments (mM). Relative expression of PPC_7190 in all treatments was normalized to average PPC_7190 expression in the 0+0 nitrate and ammonium treatment. All values represent the average actin and ubiquitin FPKM normalized $\log_2$ transformed counts. Treatments consisted of modified Hoagland's solution with varying amounts of nitrate and ammonium (mM) and a deionized water treatment (diH_2O) control. Plots show the mean values with error bars indicating ± SEM (n = 6). Letters represent the result of Tukey's multiple comparisons test (α = 0.05).

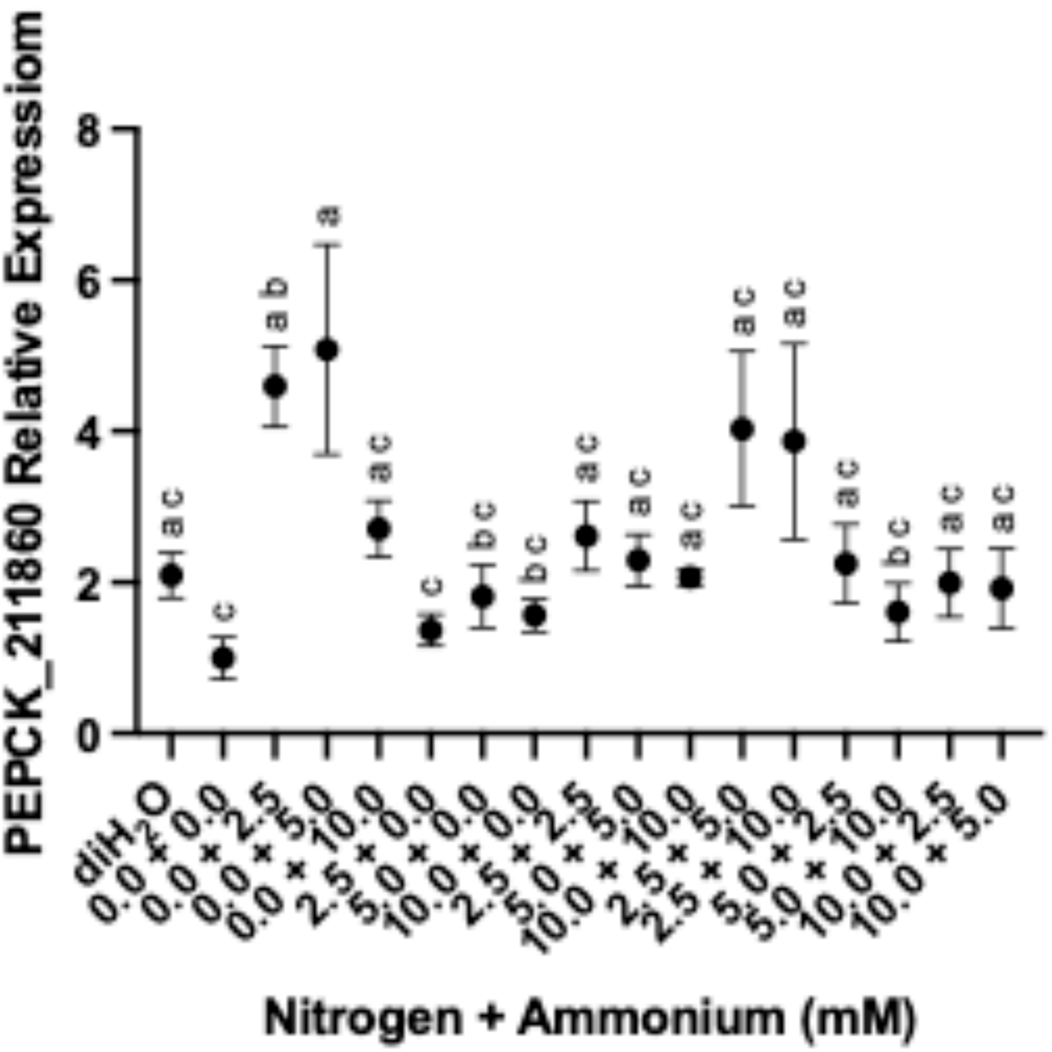

Figure S4: Relative expression of phospho*enol*pyruvate carboxylase kinase (PEPCK_211860) among nitrate and ammonium treatments (mM). Relative expression of PEPCK_211860 in all treatments was normalized to average PEPCK_211860 expression in the 0+0 nitrate and ammonium treatment. All values represent the average actin and ubiquitin FPKM normalized log_2 transformed counts. Treatments consisted of modified Hoagland's solution with varying amounts of nitrate and ammonium (mM) and a deionized water treatment (diH_2O) control. Plots show the mean values with error bars indicating ± SEM (n = 6). Letters represent the result of Tukey's multiple comparisons test (α = 0.05).

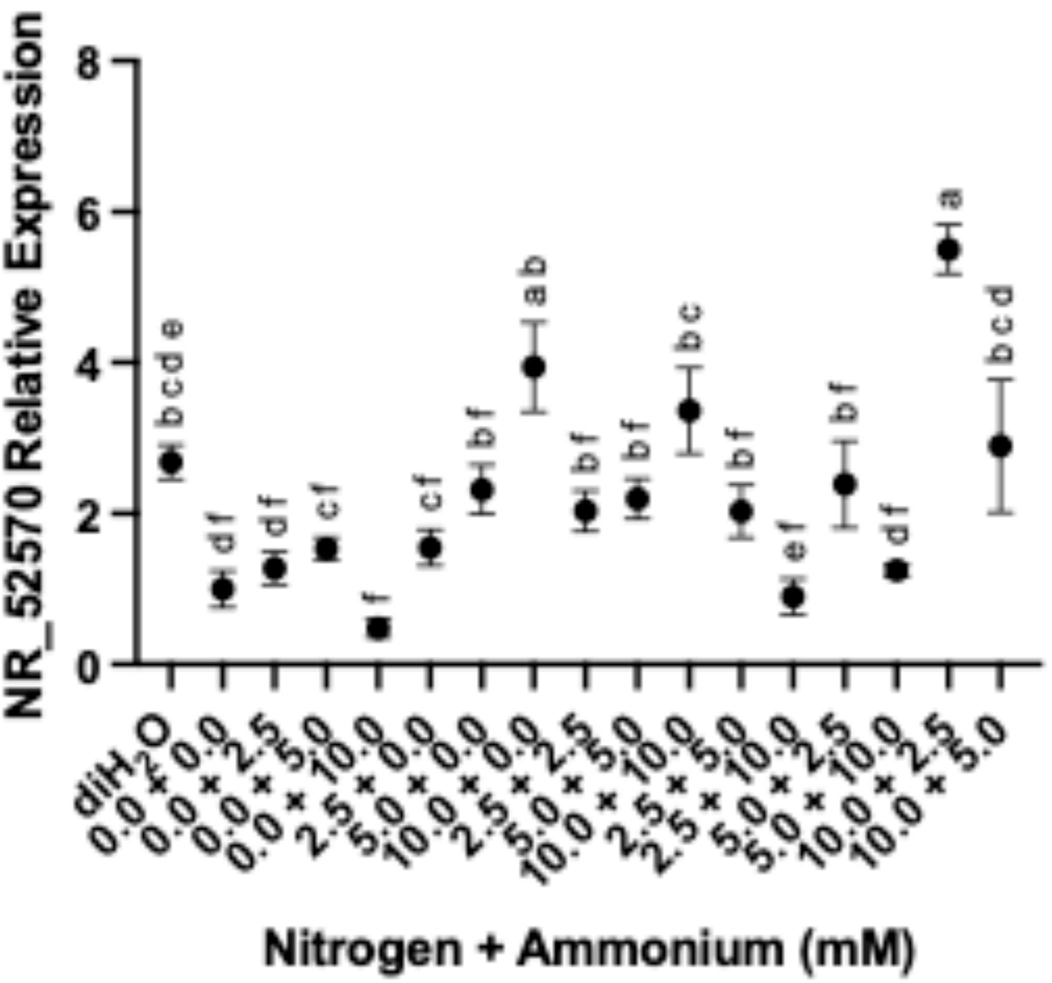

Figure S5: Relative expression of nitrate reductase (NR_52570) among nitrate and ammonium treatments (mM). Relative expression of NR_52570 in all treatments was normalized to average NR_52570 expression in the 0+0 nitrate and ammonium treatment. All values represent the average actin and ubiquitin FPKM normalized $\log_2$ transformed counts. Treatments consisted of modified Hoagland's solution with varying amounts of nitrate and ammonium (mM) and a deionized water treatment (diH_2O) control. Plots show the mean values with error bars indicating ± SEM ($n = 6$). Letters represent the result of Tukey's multiple comparisons test ($\alpha = 0.05$).

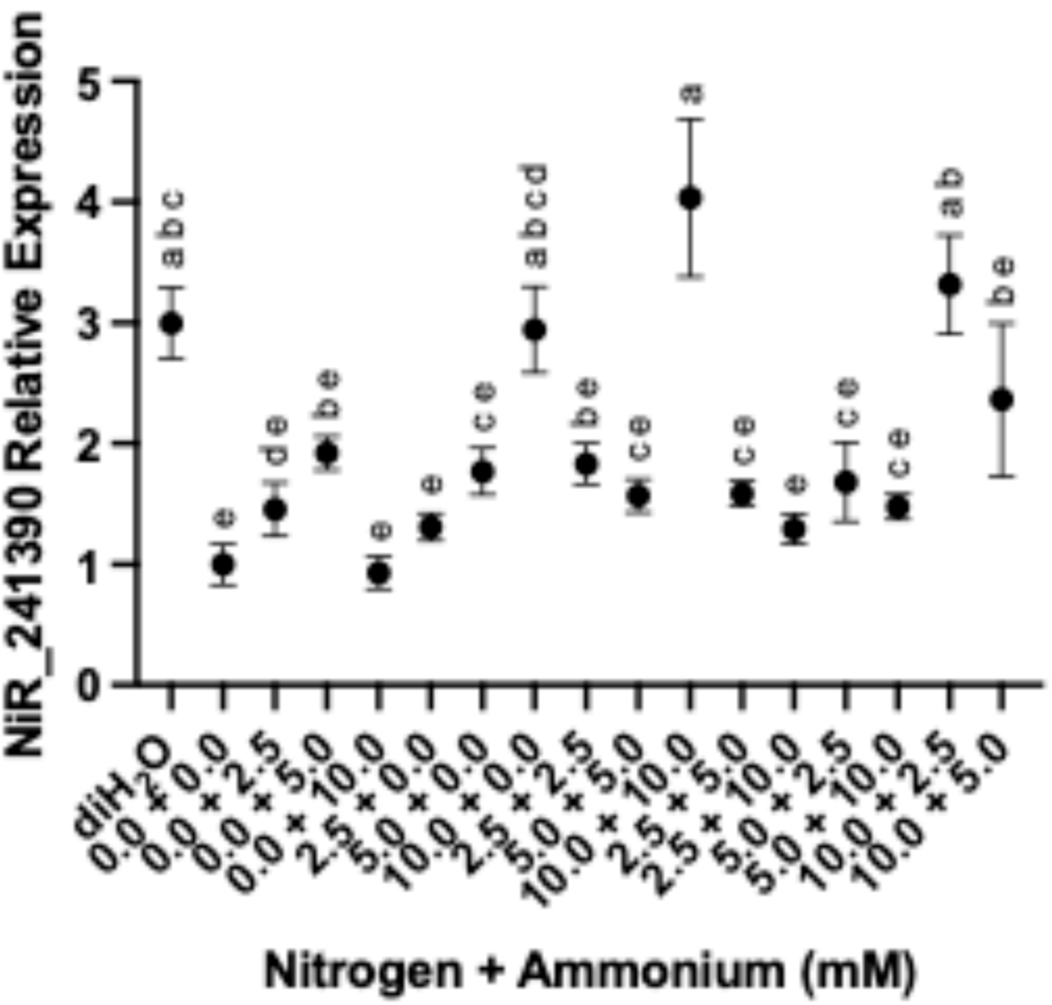

Figure S6: Relative expression of nitrite reductase (NiR_241390) among nitrate and ammonium treatments (mM). Relative expression of NiR_241390 in all treatments was normalized to mean NiR_241390 expression in the 0+0 nitrate and ammonium treatment. All values represent the average actin and ubiquitin FPKM normalized log_2 transformed counts. Treatments consisted of modified Hoagland's solution with varying amounts of nitrate and ammonium (mM) and a deionized water treatment (diH_2O) control. Plots show the mean values with error bars indicating ± SEM ($n = 6$). Letters represent the result of Tukey's multiple comparisons test ($\alpha = 0.05$).

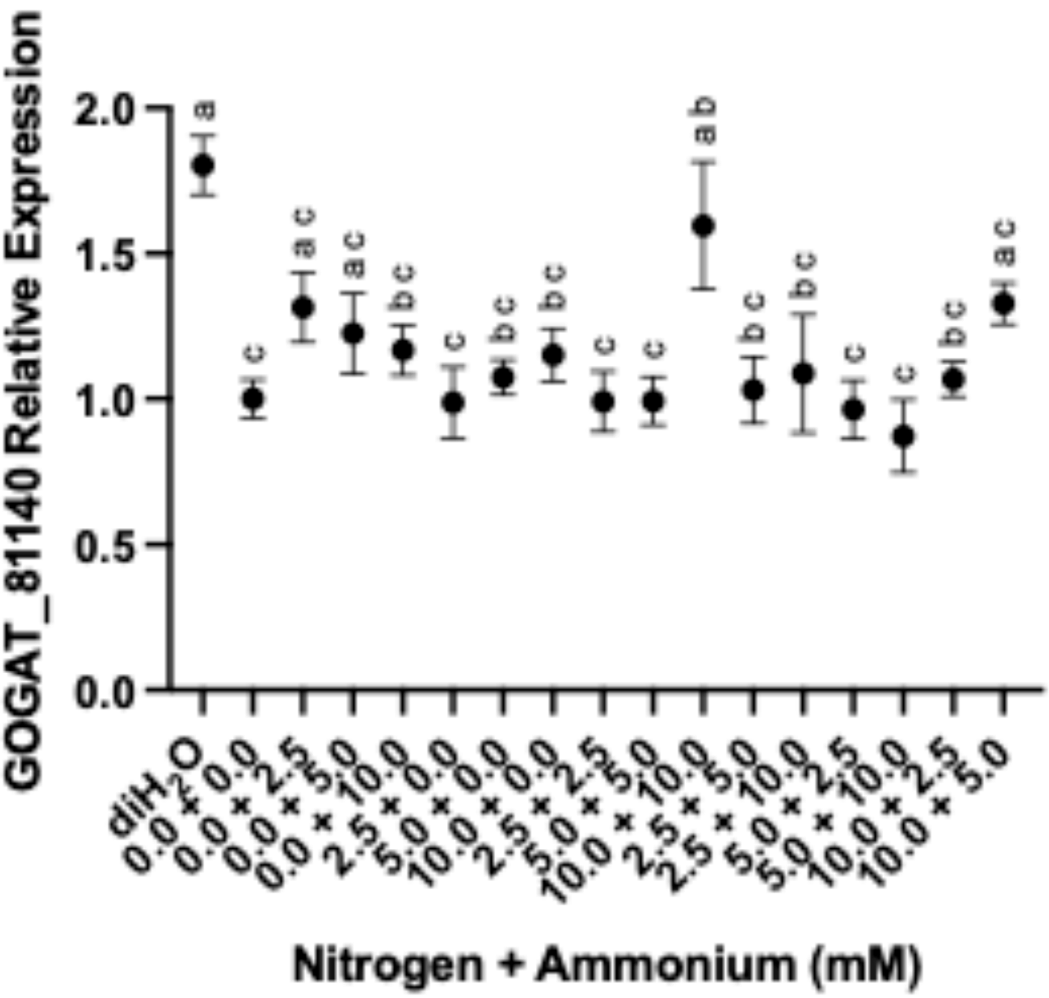

Figure S7: Relative expression of glutamine oxoglutarate aminotransferase (GOGAT_81140) among nitrate and ammonium treatments (mM). Relative expression of GOGAT_81140 in all treatments was normalized to average GOGAT_81140 expression in the 0+0 nitrate and ammonium treatment. All values represent the average actin and ubiquitin FPKM normalized log_2 transformed counts. Treatments consisted of modified Hoagland's solution with varying amounts of nitrate and ammonium (mM) and a deionized water treatment (diH2O) control. Plots show the mean values with error bars indicating ± SEM (n = 6). Letters represent the result of Tukey's multiple comparisons test (α = 0.05).

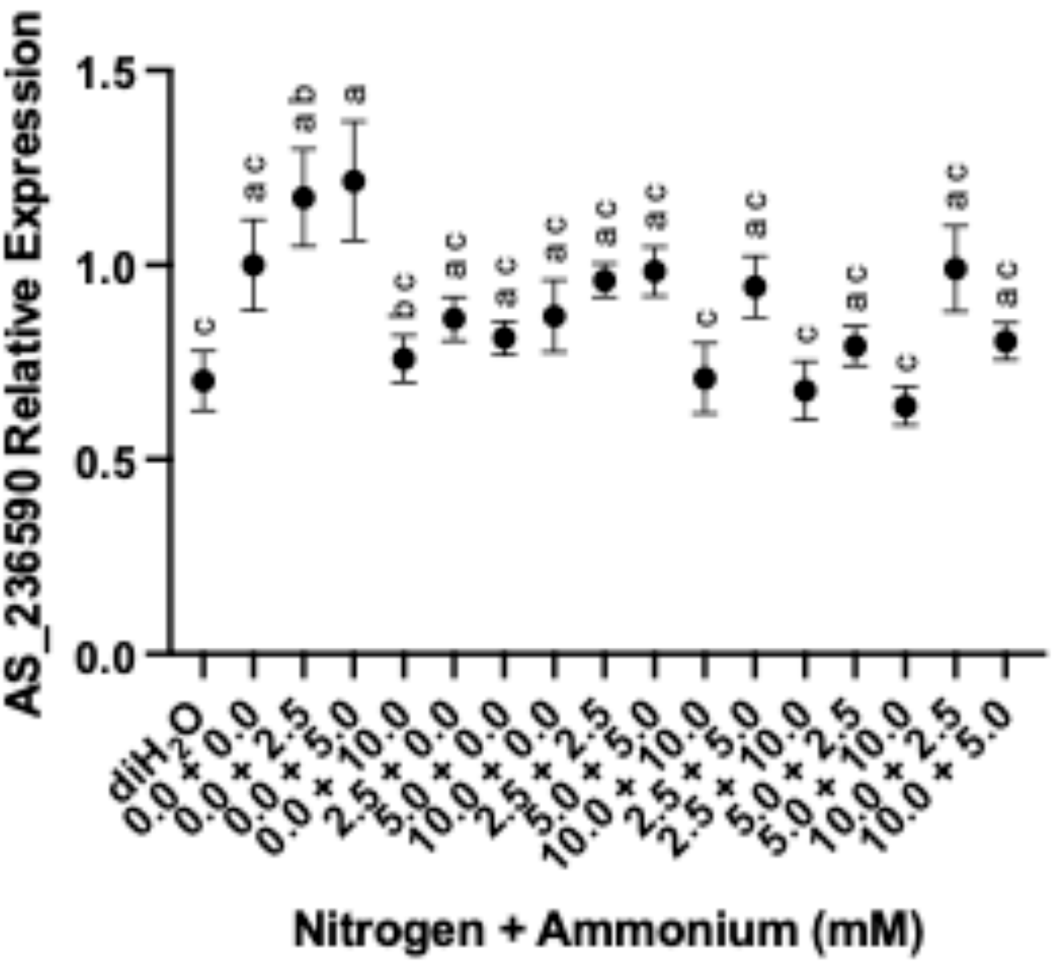

Figure S8: Relative expression of asparagine synthase (AS_236590) among nitrate and ammonium treatments (mM). Relative expression of AS_236590 in all treatments was normalized to mean AS_236590 expression in the 0+0 nitrate and ammonium treatment. All values represent the average actin and ubiquitin FPKM normalized log_2 transformed counts. Treatments consisted of modified Hoagland's solution with varying amounts of nitrate and ammonium (mM) and a deionized water treatment (diH_2O) control. Plots show the mean values with error bars indicating ± SEM (n = 6). Letters represent the result of Tukey's multiple comparisons test (α = 0.05).

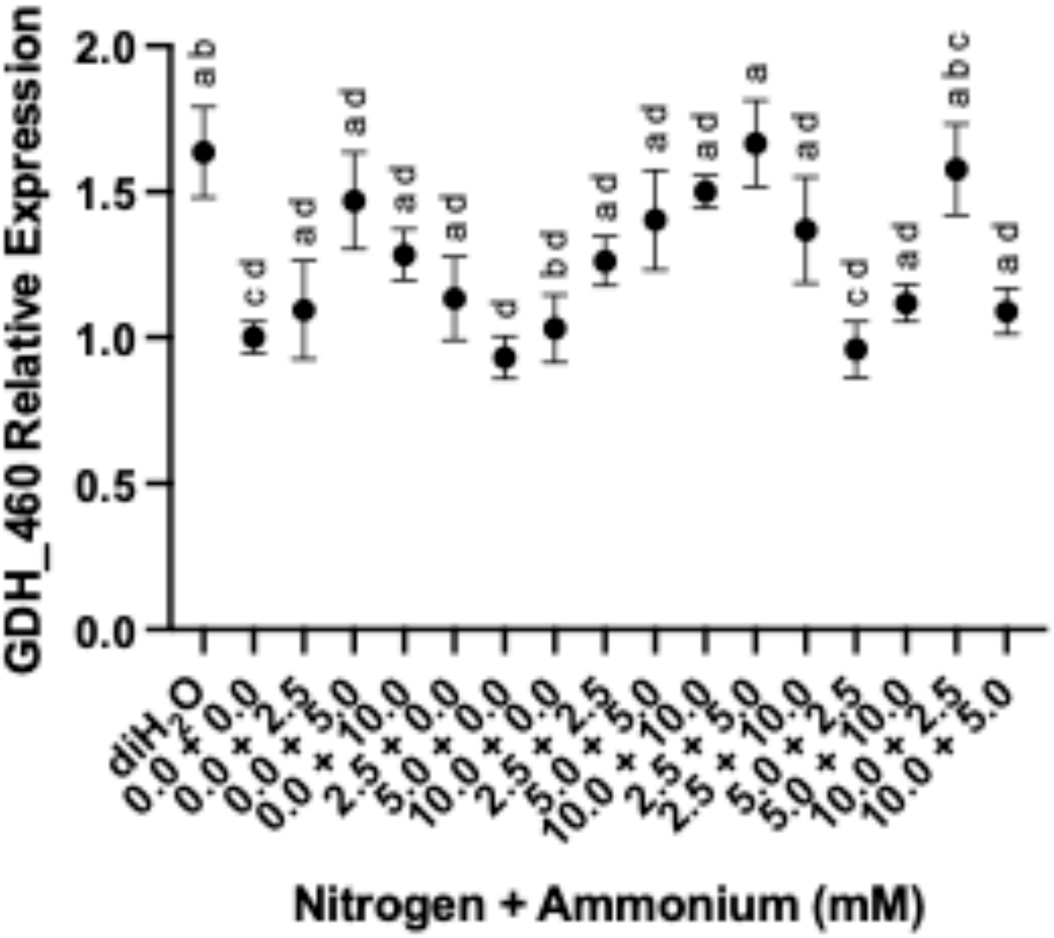

Figure S9: Relative expression of glutamate dehydrogenase (GDH_460) among nitrate and ammonium treatments (mM). Relative expression of GDH_460 in all treatments was normalized to average GDH_460 expression in the 0+0 nitrate and ammonium treatment. All values represent the average actin and ubiquitin FPKM normalized log_2 transformed counts. Treatments consisted of modified Hoagland's solution with varying amounts of nitrate and ammonium (mM) and a deionized water treatment (diH_2O) control. Plots show the mean values with error bars indicating ± SEM (n = 6). Letters represent the result of Tukey's multiple comparisons test (α = 0.05).

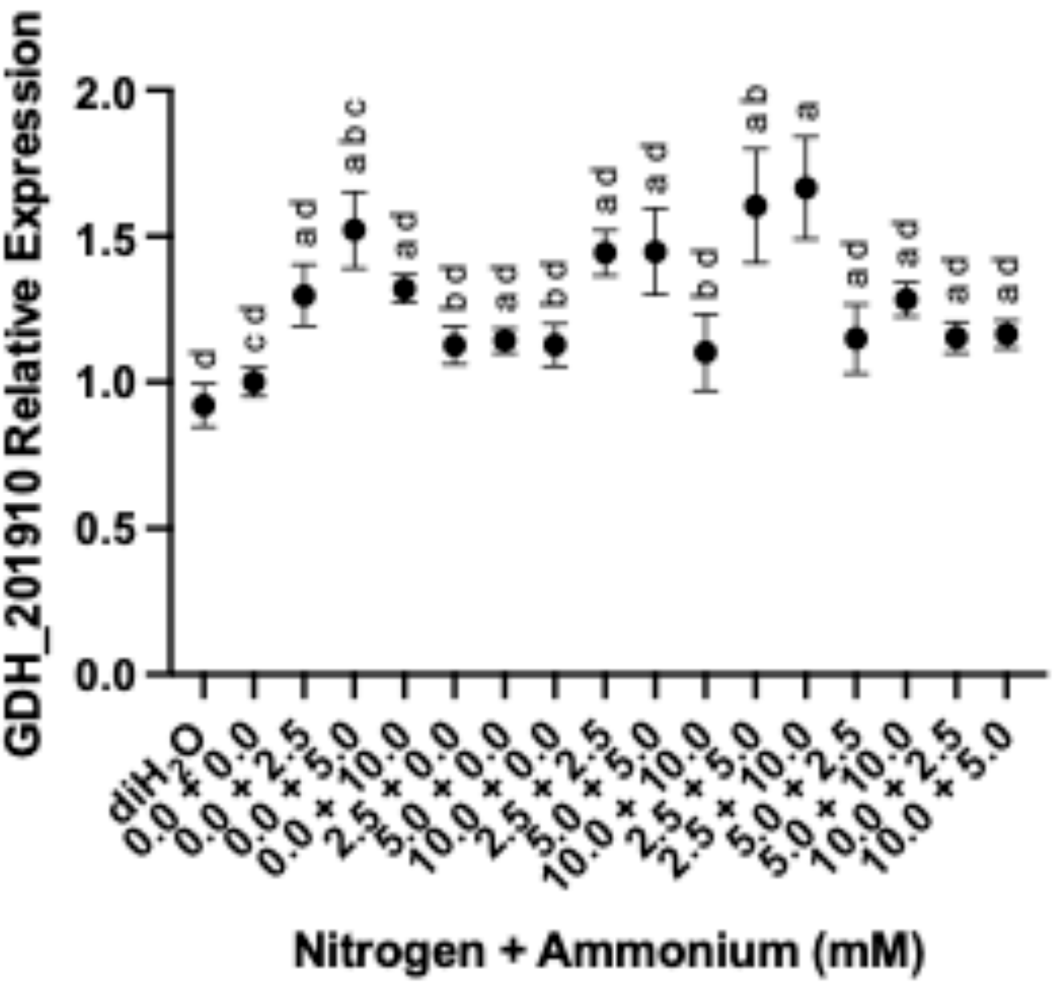

Figure S10: Relative expression of glutamine dehydrogenase (GDH_201910) among nitrate and ammonium treatments (mM). Relative expression of GDH_201910 in all treatments was normalized to mean GDH_201910 expression in the 0+0 nitrate and ammonium treatment. All values represent the average actin and ubiquitin FPKM normalized $\log_2$ transformed counts. Treatments consisted of modified Hoagland's solution with varying amounts of nitrate and ammonium (mM) and a deionized water treatment (diH_2O) control. Plots show the mean values with error bars indicating ± SEM (n = 6). Letters represent the result of Tukey's multiple comparisons test ($\alpha = 0.05$).

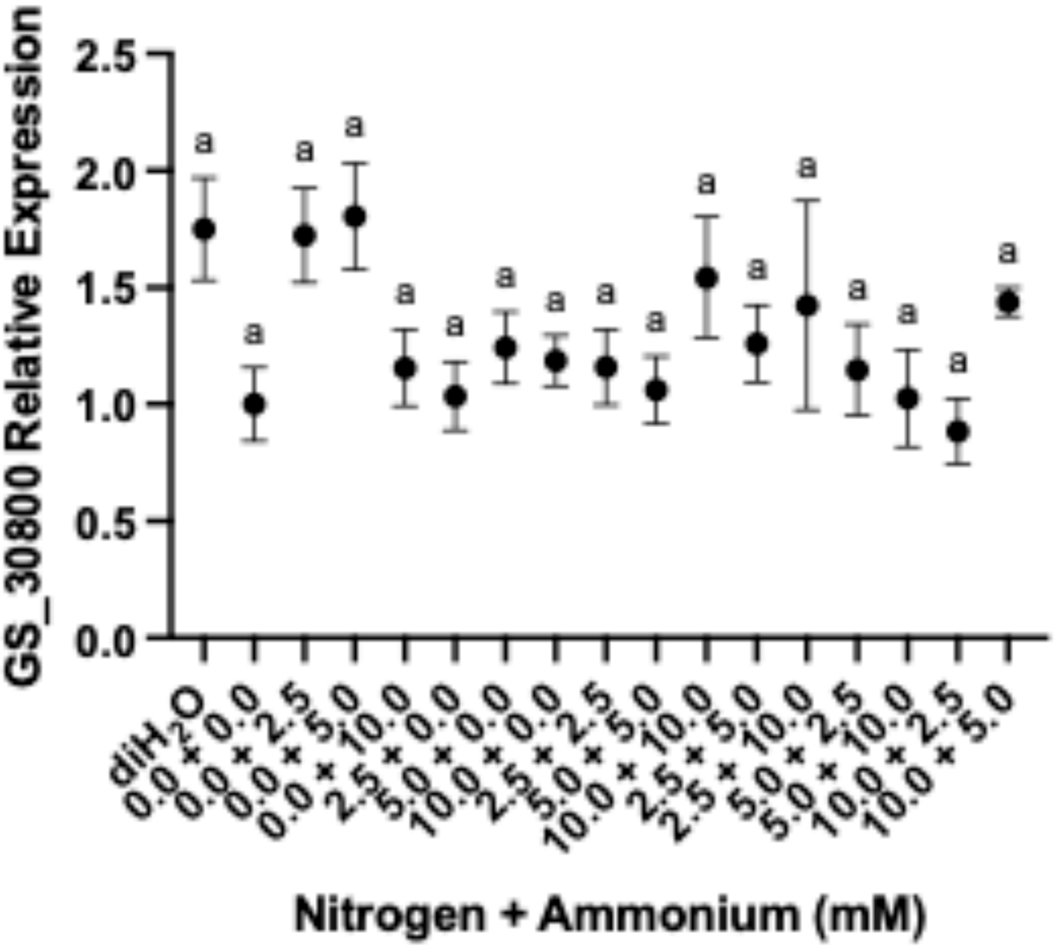

Figure S11: Relative expression of glutamine synthase (GS_30800) among nitrate and ammonium treatments (mM). Relative expression of GS_30800 in all treatments was normalized to mean GS_30800 expression in the 0+0 nitrate and ammonium treatment. All values represent the average actin and ubiquitin FPKM normalized log_2 transformed counts. Treatments consisted of modified Hoagland's solution with varying amounts of nitrate and ammonium (mM) and a deionized water treatment (diH$_2$O) control. Plots show the mean values with error bars indicating ± SEM (n = 6). Letters represent the result of Tukey's multiple comparisons test ($\alpha = 0.05$).

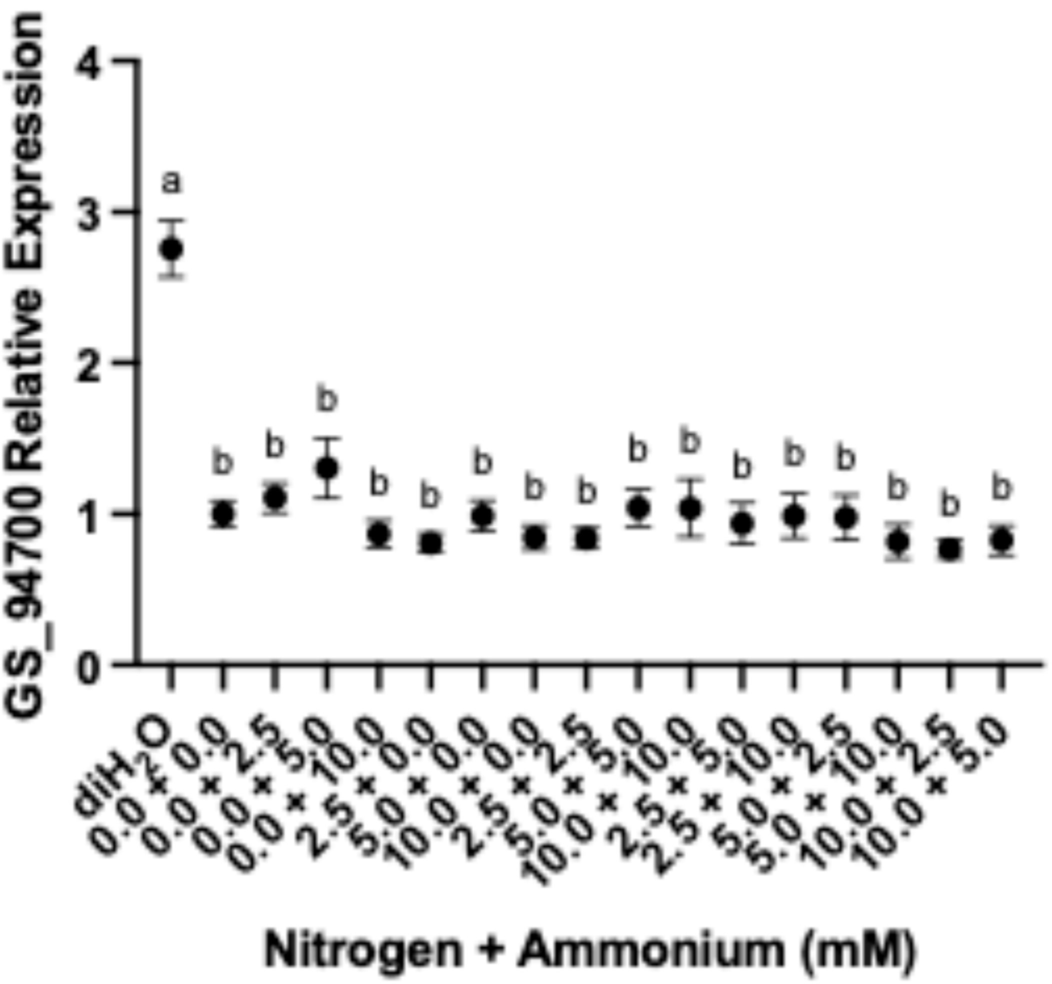

Figure S12: Relative expression of glutamine synthetase (GS_94700) among nitrate and ammonium treatments (mM). Relative expression of GS_94700 in all treatments was normalized to mean GS_94700 expression in the 0+0 nitrate and ammonium treatment. All values represent the average actin and ubiquitin FPKM normalized $\log_2$ transformed counts. Treatments consisted of modified Hoagland's solution with varying amounts of nitrate and ammonium (mM) and a deionized water treatment (diH_2O) control. Plots show the mean values with error bars indicating ± SEM (n = 6). Letters represent the result of Tukey's multiple comparisons test (α = 0.05).

Figure S13. Reference gene sequences use for gene expression studies. Forward and reverse primers used are indicated by yellow and green highlighting, respectively (see Table S3).

ALMT_206820

GTCAATGTCATCACATTATCATCAACATCTGGAAGACGAAAGACAACCCATTCTCTGATTTTTGTAGGATAATTTTCAGCCCACCTCTCTCTCCC
CCTCTTTCTCCAACCCCACCACAACCCAATTCCTGATCAATCATATTCAATTGCAAGAGTAATCGAACTACAACACTTTTTGCTTGGTGGGTTTG
TTTGAATTTGGCTAGTACATGTTCAGTCTCCAGTTAATTTTGTCGTGTTTCTTCATCTGTCTTAACTCTATCATTTCTACCCTTCAATGGGTTTTTT
AGCTTCCAGTTGAACTAAATTTGTCAATAAACACCTTCTCCTTTGGATGCTTTAAGTAACCAATCCACGTATCTATCTCTATTTGCTGCTTTTCTT
GTAAATGGCGGCAAAATTCGGATCTTTGAGGCAGAGTTTCATTGAGAAGAGCAAACGGAGATCGCTGTCCAGAAGGGGGTACTCGGAATTA
GGGTTTGCAGGGCCAGAGAATGAGGCTGATGGCAACGAGGGCGTCAAGTGTTGCTGTTTTGGATCTAAACGAGGGTGTTATCGCCGAATTG
TTGACGGGTTTTTGGGCTTCTCAAATGGGTTTAAGGATGCTATGTTTAAGTTGTATGACATGGGTCGATCTGACCCTAAAAAAATCTGCTTTGC
ATTGAAGATGGGGAGTTGTTTCACTCTGATTTCTTTGCTGATGTACCTTAAAGCGCCGTTCCAAGATATCGGCTCGTATTCACTGTGGGGTAGT
TTAACTGTGATCGTCTGTTTTGAGTACAGCGTAGGTGCATGTCTAAGTAAAGGCTGTAATCGTGCTCTGGGAACGTTAACTGCTGGAACTCTT
GCTTTTGGAGTGTCAGAAATAGCAATTCATGCTGGGGCATTAAAAGAACCAATCATTATTCTTGGCATTTTCACTTCAGGAACTTGTGCTAGTT
ATCTCAAATTATATCCGACAATGAAGCCATATGAATATGGCTACCGGGTATTCTTGATGACATGGGCCATAGTGACGATGACTGGAAATAAAA
CAACGAAATTCTTCCGTACTGCATTTTTTCGATTGATGCTTGTTGCAATTGGTGCTACTACTGCTTTTATTGTGAACGTATGTGTTTTTCCTATCT
GGGCTGGCGAGGATCTTCACAAGGCTGTCGTAAAAAATTTCAAGGGTGTTGCTGCTTCCTTGGAAGCATGTGTAAATGGGTACCTGCAGTGT
GTGGCATTTGACAAAATCCCGTCAACCATTCTTACATACCGAGCTTATGATGACCCTCTATATGGTGGCTACAGGGCAGCTGTCCAGTCTTCAA
GCAAAGAGGAATCACTGGTGGAGTTGGCAAAATGGGAACCTCCTCATGGGCGTTACCGATCATTCAAATATCCTTGGGAGAACTATGTTAAA
GTTGGTGGTGCACTGAGGCATTGCGCATTTATGATCATGGCAATGCATGGATGCATGCTTTCTGAAATTCAGGCGCCAGCTGAGAAGAGACA
AGTTTTTGCTGATGAAGTACGCAGGGTTGCTGTTGCTGGAGCTAAAGTTCTACGTGAGCTCGGAGTCAAAGTTGAGAAAATGGAGAAACTAG
ACCCTGGCGATATACTCGAAGAAGTGCATATCGCAGCTGAGGAGCTGCAAATGAAGATAGATCGGAAGTCTTATCTCTTGGTCAATTTTGAG
CTTTGGGAAGAAGGCAAGCGACCCAAGGAATTCGAAGATCCTGAGCGTGTTGCCCAGATGAAGGATTGTGGTGGAAGTAGTCATCAGCTAG
TGTTCAACTCTCTCAGCGAAGCAGTGCTGAATATCGATACTGATCAAATGCAATCAAACCTTGGGATCCCCAATTCCCGGGCTTCTATGTTCGG
TGTCGGACCTTCACTGCAAGCGATGTTGTCGACTGAGAGCATTGGTTGCCGGACTCAGTGGCCTTCCCGTCTTTCAGTTCTTCCGGACCTCACC
ATCAATGAAAAGGAAGCAAAAACATATGAGAGCGCGAGTTCTCTTTCATTGGCAACTTTTACTAACCTTTTGGTGGAGTTTGTTGCAAGGCTC
GGGAACTTGGTGGATGCTTTTGAGGAACTCAGTGAGAAGGCACACTTCAGGGAACCAACCATAGAACCCCAAGACCAAAAGGAGACTAATG
GGTTTTGGAACAAGTTGATCAAGTGCTTTCAGTTTAAGAACTGAGATTTTGCCTAAGTTATGTTGCAGGCAGATAAATAAACAGGAAACCGAA
GGGGAGAAACAGATTGGTTCTAATTCGAAAAGATGTTCCTTGACCCTGCGTTGGCAGGATCCGTACTGGAATTTTTGTTACTGTGTATTTATA
AGTTCCCTATTCAAGTTGTAAAAAAACAATGGAATCTGTACTTTATACACACCAACCCCCCAAAAAAAAAAGAAAAAAGAAATGGAATCTGG

PPC_7190

CATCAATCTCTCTCATTCATGCCCGGAGTCATCGCAAACCCGTGATACAAGCCCCGCCCCTAAACTCGTCGACTTTGATCTGACTTGAATCAAT
AGCCACCGCAATCTCAGTCACAGGGCTGTGTTGCTTCCAAAAAAACTCGGCTTCTGCGAGAATTTAGCACTTCACTTCCACTGAGTGAAGTTCC
AACACCAAGGAGAAGAGAAAGAGTCAAGGATGGCGACTGCCAAGTTGGAGAAATTGGCTTCAATTGATGCTCACTTGAGGCTGCTTGCTCCT
AGGAAAGTTTCTGAGGATGATAAGCTTGTTGAGTATGATGCTTTGCTGCTTGATCGTTTCCTCGACATTCTTCAGTCTTTGCACGGCGAGGAC
ATCAGGGAGACGGTTCAAGAATTGTATGAGCATGCTGCTGAATATGAAAGGACACGTGATCCTAAAAAGTTGGAGGAGTTGGGAAACATGA
TCACCAGTTTGGATGCTGGTGATTCTATTGTGGTTACAAAATCTTTTACTCACATGCTTAACTTGGGCAACCTTGCTGAGGAAGTCCAGATTGC
TCATCGCAGAAGGATCAAAAAATTGAAGAAAGGAGATTTTGTTGATGAGAGTTCTGCAATAACTGAATCAGATATTGAGGAAACACTAAGGA
GACTTGTAGTGGATTTCAAGAAATCTCCTCAAGAAATTTTTGATACTTTGAAAAACCAGACTGTTGACTTGGTTTTTACAGCTCATCCTACACA
ATCTGTTCGCAGATCTTTGCTTCAGAAGCATGGAAGGATAAGGGATTGTTTGACCCAATTGTATGCCAAAGATATCACCCCTGACGATAAGCA
GGAGCTTGATGAAGCTCTTCAAAGAGAGATACAAGCTGCATTCCGTACAGATGAAATCAAGAGGACTCAGCCAACTCCACAGGATGAGATG
AGGGCAGGGATGAGTTACTTCCATGAAACAATTTGGAAAGGTGTTCCAAAATTTCTACGTCGTGTTGACACGGCATTGAAGAACATAGGGAT
AAATGAGCGTGTTCCCTATAATGCTCCTTTAATTCAATTCTCATCTTGGATGGGTGGTGATCGAGATGGAAATCCAAGAGTAACTGCTGAAGT
TACTAAAGACGTATGCTTGTTGGCCAGAATGATGGCTGCTGAGCTCTATTTCTCTGAGATAGAGGACCTTATGTTTGAGTTGTCAATGTGGAG
ATGCAATGATGAACTCAAAGCTCGAGTAGATGAACTTCTTAAAGATTCGAAGCAAGATTCCAAGCATTATGTTGATTTCTGGAAACAAATCCC
TCCTAATGAGCCCTATCGTGTTATTCTTGCTTATGTGAGGGATAAGCTCTACAATACCCGTGAACGTGCTCGTCAGTTGTTGTCAAAGGGGGG
TTCAACTATCCCTGAAGCCTCAACTTTCACCGATGTTGATCAGTTTCTGGAGCCTCTTGAGCTGTGCTACAGGTCACTTTGTGATTGCGGCAAC
CAGTCTATTGCTGACGGAAGTCTTCTTGATTTCATGAGACAAGTTACTACTTTTGGGCTCTGTCTCACAAGGCTTGATATAAGGCAAGAATCAG
AGAGGCACAATGATGTGATGGATGCTATTACTAGGTATCTGGAGATTGGATCCTATCGGGAATGGCCAGAAGAGAAGAGGCAGGAATGGCT
CTTATCTGAGCTTAAGAGCAAACGCCCTCTATTTGGTGCCAATCTCCCCAAAACTGAAGAAGTTGCTGAAGTATTGGACACATTTCATGTGATA
TCGGAACTCCCACCTGACAGTTTCGGGGCATACATAATCTCCATGGCTACCGCCCCTTCAGATGTGCTTGCTGTTGAGCTTCTGCAACGTGAAT
GCCATATTAAGAATCCGTTGAGGGTTGTCCCCTTGTTTGAGAAGCTTGCTGACCTGCAGGCAGCTCCTGCTGCCATGGCCTGCCTGTTCTCTAT
CGATTGGTACAAAAATAAGATTAAGGGGAAGCAAGAAGTCATGATCGGATACTCTGATTCTGGTAAGGATTGTGGTCGTCTCTCAGCTGCTT
GGCAGCTTTACAAGGTCCAAGAGGAGCTAGCAAGGGTTGCCAGGCAATTCGGAGTGAAGCTTACAATGTTTCACGGACGAGGCGGGACTGT
GGGAAGAGGTGGTGGCCCTATTCATCTTACCCTTTTGGCTCAGCCACCTAACACAGTCAATGGATCACTTCGTGTTACGGTACAAGGTGAAGT
AATTGAACAGTGCTTTGCAGAGGAGCATTTGTGTTTTAAAACACTACAGTGGTACACAGCTGCTACCCTTGTACATGGGATGCGTCCTGCGAT

CTCTCCACTACCAGAATGGCGTGCTATATTAGATCAGATGGCAGATCTTTCAACAAAGGAATACCGCTCCATTGTTTTTGAAGAGCCTCAATTT
GTCAAGTACTTCCGCCTTGCGACACCAGAGACAGAATATGGGCGCCTGAACATTGGAAGTCGTCCAGCAAAGAGAAAACCAAGTGGGGGAA
TCGAATCACTCCGTGCAATCCCT

PEPCK_211860

GTTTATTTCCCTTCTTCATTGATGAAAAATCTTTTGCAAATGGGAAAAAGGCATTAAACTTAGAATGTGATGACCCCTTCAATAAACTAAGATC
CATGAATTGCTTCAAAAATATTCAATACTATTTCAACCTCCAAGTCTCCAGAGCAATGAACATAGGATTACAGATATCTTATTGCAATCTTTTTC
GTCATCATCACTAGTAAGAAGAAAAGCATGATTACTAAGTCCACTCCGACACATATGATCTTAGAATACAGGACCAGCTGCCAGGATCTCCTC
AGTCAATTTGTTGCCCTTGCCGATCTTGTAATCCGCAAACACCTCAAAATTTTTCTTAAACAACCCTGCTAGCTTGAGTAGTGTCTCCTTGTAAG
CATCCTTGTCGGGCCAAGCGTTCACTGGATCGAGGATCTCTGAAGGCACGCCTTCAACCCCAGTTGGAATCTCGAGACCAAAGACTTGTGTCT
TCTTGTAGCCTGCATTCAAGAGGGCGCCGGAGTGTATGGCATCGATAATCTTACGGGTATATTGTAACTTGATGCGGCTTCCTACACCATACC
TTCCACCGCACCAACCAGTGTTGACGAGCCATCCAGTAGCACCATACTTCTGCATCTTCTCCGCAAGCATAGCTGCATACTTTGTAGGATGCAT
CATTATGAATGCTGCACCAAAACAAGCAGAGAAAGTTGCTGTTGGTTCTTTGATGCCATCTACCGTACCAGCCACGAGAGCTGTGTAGCCGCT
GATGAAGTGATACATGGTCTGAGCCAGGCTCAACTTGCTTACTGGTGGAAGCACACCAAAGGCATCACACGCCAAAAGGATGACATTCTTAG
GGTGTGGTCCAACACATGGTAGTTTTGCGTTTGGTATGTACTCAATGGGATATGCTGCTCTTGTATTTTCTGTTACAGACTTGTCGGAGTAGTC
TACCTCTCGGGTGTGCTCATCAAATACCACATTCTCCAGCACAGTTCCAAATTTGATGGCATTCCAAATATCAGGCTCTTTCTCCCCAGAGAGA
TCAATGCACTTGGCATAGCATCCTCCTTCGATATTTGACACACCATTATCGCTCCAACAATGCTCGTCATCACCAATCAAGTATCGGTTGTGATC
AGTTGACAGAGTTGTCTTCCCAGTACCTGACAAACCAAAGAAGAGGGCGACATCTCCATCTTTGCCCATATTGCAGCCAGAGTGAAGAGACA
GGATTCGACGCTTAGGCATGAGGTAATGCATTACACTGAAAAGTCCTTTCTTCATCTCCCCGGCATACTGGGTGCCAAGGATGACCATTTCCCT
CCTAGCCAAGTTAAGGTCTATGCTAGTAGACGATGTCATGTAGTGTGTGTAACGATTACACGGAAACTGCCCAGCATTGTATATAGTGAAGTC
CGGAGTACCAAAATTCTCCAGCTCTTCGGGTGTAGGTCGAATACACATGTTGTGCATAAACAAAGAATGGTAAGCTCTAGCAGATACAATCCT
GACTTTAATTCGGTTCTGTGGATCCCAATTCAGGAATTGGTCGTTCACAAATACCTTATCCAACGAATTCAAATAATCAACAGCTCTTTCTCTGT
TGATCATGAAGGTATGTTCATCCATCTCAATGTTTGGTGAGCCCTTGCCCCACCAAAGCTCATCCGCAGTGGTTTCATCCTTGACAACACGCTT
GTCTCTTGGAGACCTCCCTGTCTTAGCTCCTGACAGTGTCGCCAGTGCACCTGTTGAGGTTATGAACGACCCTTTCTCATACTTTATCGCCTGCT
CATACAGCTCAGCAGGAGAGAGATTGTAGAGAACGTGAGTGAACTTGAGGGCACTATCACTGACGCTGATGGTGGGGTTGACGAGGTGAT
GATCCACGTGCTTGGGAGGGTGTGCCTTCCTTGCTGGGTCTCCTTTCACCACCTTTGGTCCTGTCTCTCTTGTTAATGACGCCAACGATGCACT
GATGGATTGGAGCTGTTGTTTCTGGCGCTCCTCTTCAGACAGGGCGGCAAAAATGGCACCGACCTGAGAGTCCTTAATGGGTGTTGTCGGCG
CCGATCTCTTCTTCTGCAACGAATGAAGCTCATCGATCGTCTGAGCCTTCACCGGTACTGCACTGTCATCGTGACATATTCCGTTCCCGTTCTTC
CTCCCCCCGTTCCCGTTTCCGTTCCCGTGTGTCTGTATCTTCTTCAGCCCGTTCCCGTTTGCCAGTCCTAGTCTCGGCGAGGCTCCGTTCCCGTTC
TCGGCCATTTCTGTTCAGGAGCTTAGCCAGGTGAAACTGATAAGACAGATTACCAAGGAGAGAGTGAGAGAGAGAGAGAAAGAGAGAGAG
AGATCAATGAAAATGAAGAGAGAAATGGAGAAGATGTTGATGAGCCTTGAAGAGAGAGAGAGAGGGGTTTACGAGGATGAGTA

NR_52570

GTTTATTATATAATTTAAAAAACGACACCCACAAACAAATTTGATAAGCAAAAACGAGTAGAGACTTGTGTTCCCAGATGTTATCAATACAAG
TCAGCCCCCAAACGACAACACTTAGGTCTCATTAATCTCTACTCTCACGATCACCACAAAAAAAAAAAAAAAAAAAAAAAGGTCTCCGCATAAG
AACTACATTACAGTACAGATTAAACTAAGCTTGAAAAGAATTTTAATTAACCCTCTTCTTCTTTTTTTAATCCCCTAAAATTTAGAAGATCAAGA
GCTGTTCTTTGATATCATAACCCATCTTCTCCAAATTGGGCTGGACAGCGAACTGGATCATGGGCGGGGGCCCGCACGTGAGTGCCAGCACA
TCCTCACCCGCCGCTGGCATGTGATCTCTGAGAATACTTTCCGTGATAAATCCGGTGTCGTATTGCCACCCTTGGGCTATACTCTTCTCCACCAC
GTACCACACTTTGACCCTGTCCTTGTACTCCTCCGCCCATTCATCAAGCTCCTCTCTCAGTAGAATATCGTCCTCTGTCCGATTGGCGTACACCA
CGTACATCTCGGTGTCGTCCTCAGGATCTTTTAGTATTGCCTGCATCACCTGATAAATAGGGGTGATCCCTGTCCCACCGGCAATCATTGCCAG
CTTCTTAGCGAACTTTGTCTTCCCATGGATCGAGAAGTTTCCTCTTCCAGTGTACTCAATGTGGCCCAATGGGCCCTTCACCTCTAGGATCGAG
CCCACTTGGAGTGAGTCTAAGTATTGTGACATGACCCCTCCGTTGGGGAACCTGGGGTGCTGCCCCTTGAAATAAATCTTGACCACTAGTTCA
AAATATCCGACTTCATCGATGGATGAGGTCGGAGTGTAGGCTCTCATGCAGAACTTATCCTCGATGGTGGCACACAAGTATAAGTGTTTGCCC
ACAGGCAACCCCAACACCTGGTCTTTCGAAGGCAATGCGAATCTAAACCTCCTCACATCATGAGAGATGGAGACCTTCTCGATGAGCTTGCAT
GGGACCTTCTCACGTGGGTTCGCCAACGCCACGTTCTTAAGCGGGGCTGACTCGGTTATCGGGGCCAGGTACAGGGACCCACCGTGGACCG
AGTTTCCAGGGGTGCTCGAATCGGAGCTGTAACCCGTTGTGATCAACTCACCGATTCTGAAGTCTTCTAAAAGTCTCTTGGCCTTGTCGGAGT
GGATGGCGTCGAACTCCTCGGTACAGTCCGTGCCGGCGTTGATGAGGATGCTATCCGGGCCTCCCGGGTGGTCCTTGAGGAAGCGGGTCGC
GTCGTACACGTGGCCCTGGACGACGATCCATGCTGACTCGGCCGAGTTGTGCTTCTTCACCTCGGACATTGAGAACATCTTTGAGGTATTGTT
CAAGAACGGGGTTGAGGCCGTCCTCTTTAGGGTCGAGCCACCCGCATCGTCGAAGTGCCGCTCTCGGGCCATCCATCCGCCCGATTTGTTTCC
GGGTTGGGTCGGATGCTCGAACACGATACCGATCTCTCCCCTGTGTGGCTTGCACACGTTGGTCTTCACCCGGAACCAACAATTGTTCATCAT
CCCCATGAGATTCCAAATGAGCTTTTCGGGTTGGGTGTTAAGGGTCTCATCCCAAGCTCGCACTGCAATCTCTTTGGCACCAAGCAAGTCCAG
CACCTCCACCTCCAATGACCAAAAACACCAACACCAGAATTTTCCATACTTGTTTCCTTTCTCAGGGTGGTCCAATTTGCAAACTTGCCATGTTT
CTCCTCCGTCCATCGTCACTTCCACTCGTGTCACTTTTTTACCTCCTCCAGAATAGGCATAGCCTCTCATGGTATAAGGGCGTTGGGTAATCCAT
GCATTGATGGGCAAGATCTCCTCGTGGCATGGCGTTGTTATCACCGAGTTTATGTTTAACTCATTGATAATGTACTCCGGCTTGTACCACCATG
CTTCAGAATTAGCAAGTTCGGCATCAACATGGGAAGGGAGGACTCTGTTATCCTTATAATGGTAGTAGTTATCGGACTCTGTGGTGGTGACA
ATGATCCTCTTGAGCCACTTGACCATCCGGCCACCGATGAACCCCGGTATGATCATCCGGACCGGGAACCCATGATCCGGAGTCAACCGCTCA
CCATTTTGCATGTAGGCCAAGATAATATCCCGGGCCGGGTCCATGGCAAACTCCTTCTTGATACTGGTCCCATACTTGGACCCGCCTCCTCCAG
GCAGGTCCTCGGCGCCCTCAAAGCACACGTTCAGGGCGCCCTTCTTGGAACTCATAATTCCGCACCTCCTAAGCACGTCCCTTAGCGGCACTC

CCCTCCACACTGAGGTGGACACTGCAGCTGACCCCCAGTTGAACCCTATGGTCTGCTTGACCAGGTTCTGCTCCTTGCGTCGGTTCCCTGCGCA
CACCAGTGTGACAGGGAACTCACGGCTCTGAAACTCGTTCACCAGTTGGTCCATGGTGATTCGGGTTGGTCGTTTGACCAACCCGGTCACCTC
TATGGTCCAGTCCTCCCACCTGGCGTTGGGGACTGGACCGTGGTTCCGCACATAGTGCAGCGGAACCGGGGTAATGAACCCATGGTGCATCA
AACGAGTCAGGGGAGGCTCCGAATTAAACGGGTGCTTTCCCGTCAGGCGGATCATGGACGGGTTCCTCTCAATCCAATGATCCGCGGTCCCC
TCATCTCGGCTGTCCTGAACGGACGGCTCTAACTCGTTGCTCCCTTTTTTGATCAACTCCTTCAACTCGTCGATCATCTCTTTTTCGTCATCATCA
TCTTCGTCACTGGAACAATACTTATTCCCATTTTCCTTCAGGTCGGGTTGTTGGACCTTCATGTAGGAAGTCGGGTTGTTGTAGGTTCGAATGG
GTGAGTTGGCACGGTGGGCGGAGAATGCGGGGTGGACGACGCCGTTTAGGCCGTTAGGCTGAAGGCGATACTGTCTATCCACAGAGGCCG
CCATGGAAGGAAGAATGGAAAATATACAAGGCAAGAAATTTAATTACGAGGGTACGGAAGAAAAAAGAGAAAAAGATTATAAAGATTAAA
GAAAGAAGACTAAGCTTCTTTCTCTTTCTTCTTCTTTTTCTTCCTCAATTTCTGTGGGTGTTTTGATATGCCAGTTTTTAT

NiR_241390
GGGCACAATTAATGCAGAAAATAAAAAACATGACAAATTGGCTACTACTAGTATTTTTTACCTACAGTTCAGCCTCCTCCATTTCCCTTGGAAC
AGCGCCAAACTGGTTGATCAGAAGGTCCACAACAATAGGCACCAAGTCCTTACATGGCACCGCCTTCTTGTAGACATCGCCTAGGTGCGAATC
ACTCCCAATCCGACCTCCCACATAGACGTCAGCACCTTCACAGGGTTTCCCATTTTCATCGCGAGTCATGCAACCCATGAAACCGATATCTGCC
ACCTGGATCTGTCCACAACTGTTGGGGCACCCAGTCCAGTGCATCCTGATTGGCCGGCTCACAGACACGAGCTTTTGAACCTCCTCGGTGACC
TTGAGTGCTCGGGCCTTGGTCTCAATGATGGCCTGCCCACAGAACTGGTTCCCTGTACAAGCCACCAACCCCCTCATCAAGATGGGTGGCTCA
GGTGAGAACTTGCTTAAAAGTGGCTCCTTTAGGAGGGCTTCCACCTTGGAGTTGTCCACATTGGGGATGATTATGTTTTGCTCAACTGTGAGC
CGGAGCTCACTTGATCCATACTCATCAGCCAACCGGGCAAGCTTGTCCATGTCATCGGCCTGGACCCGGCCCACAGGAACGTGGAGGCCCAC
AAAGCTTAGGCCTTTTTGTTTCTGAGGATGGACACCCAAGTACTCTCTCCTCTGCCATTGTTTCTGAATCAAGTCCTCGAGTGCTGCTCTTTCGA
GTACTTGGTTTGGCATCCTCTTCTCCACCTCCGCTCTGAATCCTTCTATGCCAAGCTCATCGATAAGCCACATCATTCTAGTCTTTTGCCTGTTTC
CCCTACTGCCGAGGTCCCTATACGCTTCAAGAACAGCTTTGCAAACTGGGATGACATCGTCCGTTGAGACCCAAGCATCTAGTGGGATTGCTT
CTTCACATCTTTTTGGGCTAAAGAATCCCCCGACCAGCAAATTAAACCCAAAGATCCCGTTCTTTACAGCAGGTATGTATGCAAGATCGTTGAT
GTGAGGGTGTTCGTAGAGATCATGGGAGCCAATGACACAGACGTTCCACTTCCTTGGCAGGTTGGTAAGAGCAGTATTTCCACGCGAATTGG
CAGTGGCAAGCTGAGACAGAAGATTAGCATAAGGGCGTGTATCAACGATCTCATGAGGGTCAATCCCAGCAAGGGGGTTCCCCACAGGGTT
ACGAATGTTGTCCATGCCACTCTGAAGACTTGTAAGGCCAACTGCATGGAGCCCTTTGATGATGTCAGGCACATCAGACAGAACCACTCCTCG
GATCTGCCAGTTTTGCCTCGTAGTCACATCTGCACAACCGTCCTTACCATACTTCCTTATAACACTAGCCAAGTACCTTGTTTGCTCGCTCGTTG
TCACCCCATTTGGTAGCTTCAGCCTCATCATGAATCTCCCATAGTGGTGTTTGCGTCGATGGAAGAGGCCAAGCCATTTGAGTCTGACATCAAC
ATCATCCTTAGTATGCTTAGACTTGTCAATCTCCTCCATTGTCAAGTGGGCCAGTTGTTGAATCCCATCTTCACTGAAGAGCTTCATGGGGTCCT
TCTCAATCTTGATCTTCTCCTGTGGGTTTATCCCTTGCCTGAACTTCTCCTTTAACACCCAGTACCCTTCTCTCTCCTCCACTCGCACCTCCAGCCT
CGCCGCGTCCACCGCCGTCGTAGCCACCGCTGCCTCGGCTGTCTGAGACTGCTGGCACCTGATGATCCTCATCATGTCTCTCCTCCTACTTCTT
GATGATGATGGGATCAAGGACAAGGATCCCATGTTGGCTGTAAATGAAGCCATCTTTTTGGTCTAACACTGGGTGTGGATCCTGCCTCTACTG
TATTGGAAAGAGCCAAATACAGGTAGCTTTACTTCAATGCGCAGAGAGACGGATCCCGAAAGACTGATAAGAACTTGGATAGTTTTGGAATC
GCAAATATACAGATACAAACAATTCTACACCAGTAAACAAAGAGACGAAAAACTCATAAGATGTGGGATAGTTTTCCTTTTTTGGGAGGGGT
GTGCGTCTAGTAATTTGATGCGTAGCTGCCAAGGAAAGGAAGTGGAGGGGTAGGCACTAGGCAGAATTCACTGAAAGTGGGCAAGTATATA
TGTAAGAGAGCAGAG

GOGAT_81140
CGACGATCTCTTCTGGCCCTGCTACGCTCTTTTGCTAACCACACCCGCAATAAAACAAAGCCAAGGGGACGAAACTCGCGAAAAAGCAACAG
AGATACAATCATATCTTACAAGCAGAGAGAAACCAGAACTTCTGCTTTCTACCATTCTATCCTCTCTCTTCTCCGCACACTCCCTTCCTCTCTCTC
TACATCCTCCATTGTTGCTGCGTCTTCAAGCTAATTGATTAGTTCCTGCCTCATCATCGTCGCGAAGGTTCTTCGAGGTTCGTCCGTCCATCAAT
GGCGCTGCAATCGGCGCCGAAGCTTTTGTACACTCCGAACTCTGTCTTCTCCGCCAACGACCGTCTTGTGTTCGCTGACTTCCTCGGCCTCTCC
AAGCGTCGCTTGCCTCGGCGAAGACTCTCTGCCGCTGCTTCCAGGAGGTTGTCCTCTTATACCGCCGGAAACTTTGCTGTTTCACCGGCGATCA
AGGCCGTCCTTGACCTTCAAAGTGGAAATGGCGCCGCCTCTTCTAGAAAGTCCAGCTCTGGTCAAGGCCTCCACCAGCAGGTTGTGAATTTGG
AGGATATACTAGCAGAAAGAGGAGCCTGTGGTGTTGGATTTATTGCCAATTTGGAAAATAAGGGATCACATCAGATTCTGAAGGACGCTCTT
ATTGCGCTTGGTTGCATGGAACACCGAGGTGGATGTGGAGCAGATAACGATTCTGGTGATGGTTCAGGCGTGATGACTGCGATTCCCTGGG
AGCTCTTTGATCAGTGGGCCAAAGAGCAGGGACTTGGTTCCTTCGACAAGTCACATTCAGGAGTTGGGATGGTTTTTCTCCCTAAAGATGAGA
CTTTTGCTGAAGAAGCTAAAAGAGTCATTGTGAGTACTTTTGCTCAGGAGGGTGTTGAGGTAATTGCATGGAGGTCTGTCCCGGTAAATGTTT
CTGTTGTCGGTCGCAATGCAAAAGAAACCATGCCCAACATACAACAAGTTTTTGTCAGAATTGTCAAAGAAGAGAGCATCGATGATGTTGAA
AGAGAGCTGTACATTTGTCGGAAATTGATCGAAAAAGCTGCTAGCGCTGAACCGTGGGCGAGTGAGCTTTACTTTTGCTCCTTATCGAATCAG
ACTATTGTTTATAAGGGGATGCTTCGTTCTGAAGTTCTGGGAATGTTCTATGCTGATCTTCAGAATGAACTTTATACATCCCCGTTTGCAATTTA
CCACAGGAGATACAGCACGAACACTAGTCCTAGATGGCCTCTTGCCCAACCAATGAGGTTTCTTGGTCATAATGGAGAGATCAATACCATACA
GGGCAACCTGAACTGGATGAGATCACGGGAGACGTCATTAAAGTCCCCTGTTTGGCGGGGTCGTGAAAGTGAAATTCGTCCTTTTGGAAATC
CAAGGGCATCTGATTCAGCAAATCTTGATAGTGCAGCGGAATTGTTGATAAGAAGTGGTCGTGCCCCAGAGGAAGCATTGATGATTCTCGTC
CCAGAGGCATACAAGAATCATCCTACCTTGATGATTAAATACCCTGAGGTTGTTGACTTTTATGACTATTACAAGGGGCAAATGGAGGCTTGG
GATGGACCTGCATTACTTTTATTCAGTGATGGGAAAACTGTTGGTGCTTGTCTTGACCGAAATGGTCTTCGTCCTGCCAGATACTGGCGCACAT
CAGACAATGTTGTGTATGTTGCATCTGAGGTTGGAGTCTTACCGATGGATGAATCAAAAATCACAATGAAAGGACGTCTAGGTCCAGGGATG
ATGATATCAGTTGATCTGCCAAGTGGTCAGGTTTATGAGAATACAGAGGTGAAGAAAAGAGTAGCATCATCAAATCCTTACGGGAAATGGGT
GAAGGAAAATTTACGTTCGATGAAGTCAGTGAACTTCCTTTCGTCTGCAGTTCTGGAAAGCGATGCAATCTTAAGATATCAACAGGCATTTGG

CTACTCTAGTGAGGATGTCCAAATGGTGATTGAAAGTATGGCTGCCCAAGGAAAGGAGCCAACATTTTGCATGGGAGATGATATCCCTCTGG
CAGTCTTGTCGCAGAAGCCACACATGCTTTATGATTACTTCAAGCAAAGATTTGCTCAGGTTACAAATCCACCTATCGATCCTCTAAGGGAAG
GCTTGGTCATGTCTCTTGAGGTCAATATTGGGAAGCGCAGGAATATATTAGAGGTTGGACCTGAAAATGCTTCACAGGTTATTTTATCCAGTC
CGGTACTAAATGAACGGGAGTTTGAGTCTTTGCTAAGTGATCCGTTGCTAAAGGCTCAAGTTCTTCCGACCTTCTTTGACATACGGAAAGGAA
TTGAAGGCTCTCTAGAGAAAACGCTTAATAGGCTCTGTGAAGCTGCTGATGAAGCTGTCAGAAATGGTTCCCAGCTGCTTGTTCTGTCAGACC
GTTCTGATGAGTTGGAACCAACACGACCTGCCATTCCAATACTCCTTGCTGTTGGTGCTGTTCATCAGCATCTTATTCAGAATGGCTTAAGAAT
GTCTGCTTCTATTGTGGCTGAAACTGCTCAGTGCTTCAGCACACATCAGTTTGCTTGTCTAATTGGTTACGGTGCAAGTGCTGTGTGCCCATAC
TTGGCATTGGAAACATGTCGACAGTGGCGGTTAAGCACTAAAACTGTGAATTTGATGCGAACTGGAAAAATGCCTACAGTTACAATTGAGCA
GGCTCAAAAGAACTTCCGCAAGGCAGTGACCTCTGGGTTGCTTAAAATCCTTTCGAAAATGGGTATCTCTCTGCTCTCCAGTTATTGTGGCGC
ACAAATATTCGAGATATATGGATTGGGCAAGGATATTGTTGATATGGCTTTCTGTGGTAGTGTATCTAGTATTGGCGGATTGACTTTTGATGA
GCTTGCAAGAGAGACATTGTCATTTTGGGTGAAGGCGTTTTCTGAGGATACAGCCAAAAGGCTAGAAAATTTCGGATTTATACAATTCAGGC
CTGGAGGGGAATATCATTCAAACAACCCAGAGATGTCAAAACTGCTGCACAAGGCAGTCCGCAACAAAAGTGAAAGTGCATATGCAGTCTAC
CAGCAACATTTGGCTAATAGACCTGTCAGCGTGCTTCGTGATCTATTGGAGTTCAAAAGTGACCGTGCACCTATTCCTGTGGGTAAAGTTGAA
CCTGCTTCATCTATTGTCCAACGCTTCTGCACTGGTGGGATGTCACTTGGAGCTATCTCCAGGGAGACTCATGAAGCGATTGCCATTGCGATG
AACAGATTGGGTGGAAAATCAAATTCAGGAGAAGGTGGTGAGGATCCAATTAGGTGGCGTCCCCTGACAGATGTGGTTGATGGCTACTCAC
CCACCCTGCCTCATCTTAAAGGCCTCCAAAATGGAGATACTGCAACTAGTGCCATCAAGCAGGTAGCATCCGGGCGTTTTGGTGTCACTCCAA
CTTTTTTGGTTAATGCCGATCAATTAGAAATCAAAATTGCTCAGGGTGCTAAGCCTGGTGAAGGGGGCCAACTACCTGGAAAGAAAGTTAGT
GCGTATATTGCCAGACTCAGGAACTCAAAACCTGGGGTTCCTCTTATTTCTCCACCTCCTCATCATGACATTTACTCTATTGAGGATCTTGCACA
GTTGATTTATGACCTACATCAGATAAACCCAAAGGCCAAAGTATCGGTGAAACTTGTAGCAGAAGCGGGAATTGGCACTGTTGCCTCAGGTG
TCGCAAAGGGTAATGCCGATGTTATTCAGATATCAGGGCATGATGGTGGAACTGGAGCCAGCCCAATTAGTTCCATAAAGCACGCTGGTGGT
CCTTGGGAATTAGGACTTACAGAAACTCACCAGATCTTGATAGAAAATGGACTTCGAGAAAGGGTCATTCTCAGGGTTGACGGTGGCTTCAG
AAGTGGAGTTGATGTCATGATGGCTGCAGCAATGGGTGCTGACGAGTATGGTTTTGGTTCTCTGGCAATGATAGCAACTGGTTGTGTTATGG
CTCGCATTTGCCACACAAACAACTGTCCGGTTGGTGTAGCCAGCCAGAGAGAGGAACTACGTGCCCCGCTTTCCTGGTGTGCCTGGTGATCTTG
TTAACTACTTTTTATATGTTGCTGAGGAGGTAAGAGGCATGTTGGCACAGTTGGGCTATGAAAAGTTGGATGATGTAATTGGAAGAACAGAC
CTGTTGAGACCAAGAGATATCTCATTGATGAAGACTCAACATCTTGATCTGAGCTACATTCTCTCTAGTGTTGGTCTACCAACTATGAGCAGCA
CTGCTATCAGGAATCAACAAGTTCATACTAATGGCCCTGTCCTGGATGATCAAATACTCTTAGATTCCGAGATTGCAGATGCCATTGAGAATG
AAAAAGTTGTTAACAAAACCTTTAAGATATATAATGTGGACCGTGCTGTTTGTGGGCGCATAGCAGGTGTTGTTGCCAGGAAATATGGTGAC
ACTGGTTTTGCTGGACAGCTCAACATAACCTTTACAGGGAGTGCTGGCCAGTCTTTTGCATGCTTTCTGACACCTGGAATGAATATTCGCCTTG
TTGGTGAAGCTAATGACTACGTTGGAAAGGGTATGGCTGGAGGAGAATTGGTTGTTGCTCCTCCGGAGAACCCTGGCTTTTGCCCAGAGGAT
GCTACTATTGTTGGTAATACTTGCCTGTATGGAGCAACGGGAGGACAAGTCTTTGTTAGAGGTAAAGCTGGTGAGCGTTTTGCGGTAAGGAA
TTCACTCGCCCAGGCGGTGGTTGAGGGCACTGGTGATCACTGTTGTGAATACATGACCGGTGGTTGTGTTGTCGTGCTTGGAAAAGTGGGTA
GAAATGTTGCTGCTGGTATGACTGGAGGTTTGGCATATATGCTTGATGAAGATGACACTCTCATTCCTAAGGTAAACAAGGAGATTGTGAAG
ATCCAGAGAGTGACTGCTCCTGTTGGGCAGATGCAGCTTAAGAGTCTAATTGAAGCGCATGTCGGAAAAACCGGAAGTTCCAAAGGAGCTTC
CATTCTGAAGGACTGGGACAAGTATCTGCCGCTGTTCTGGCAATTGGTTCCACCAAGTGAAGAAGACACTCCAGAGGCTTCTGCAGCTTACCA
GGAGGAGGCCGCTGGGCAAGTCACCTTACAGTCTGCCTAGTCAATGGACCTACACGAGAGAGAGAGAGAGAGAGGGGAAACCTTTCGCCTCGC
TTTCACCTCTTAAGCATTCCGATCCGCTCAAAATCACGTTCC

AS_236590

CTCGTATATTTGCCAAGGAGGCGCATTGTTTGCATTCAAGGCATGTCGCTTATCAAGGCCAAAGTTTTAACGGGAAGTAATACATGACCAGTG
TAAGGTCAGCCAAAAAGAGAGCATCGAAGAATGAAACGAGAAATATTTCACCAATCATAGAAAAGAAATATTTACTGGAGGCAAGAAGCAG
CTTAAGTTACAACATGAAGCCAACTGTATTCAGCTTTCATCACTGAAGATCACAGAATCCAGGCTCAAGGTGAAAGCCAGAAAGACAACCAA
AAGCAAATCTAATCTGCCCATTCTGATGTTAGGAAGTAAGATGCTATTTAGCACTACCAAAGTGCTGAAAATAACCCCTGCACAGCTTCCTTAA
TGTGATCGCTGGAAATACAGCAATATCAGATTGCATCTCATAAATGAACGTCAGCCCAATCCTCAGACGACTGTGGCAGTCTTCCCCACAATTC
CATCTTGGAGTGCTTTGAGAGAGTCGGATACTGGAGTAACATTGTTAGCCTCTCGTGCTTCCTCATAAGCAGCGTCATGTACCCCAAGGGCAG
CACGACCTGATGGATCAAGGTTTTTTGACCAAGCTGCATCCCACTCCACCGCTTTTGCAGTACTGCAGGCCACACTTGGACCTCCTGGAACAGT
TTGTCTAGCAGCACTCTTGGGGAAGAATTTCTCAAAGATTGCTCTGTAATAATATGCCTCTTTTGTAGTTGGAGTGTTTTCAGGGTAAACAAAA
CTGGCATTTGTCAACATTGCATCTGTAACATGTTTGCTCGCATGATCCTTCAAACCATCAATCCAACTGTATCCCACTCCGTCACTGAACTGTTC
CTTCTGCCGGTACAATATATGCTTGGGTAAATATGGTTTTTTATCATCATCAAAGGCATTGCGTAAAATCCACTTCTCAATCCTTCCTTGGTCAC
GTTTGATCATTTTCCACTCTGGATCAATGCTCATTGCAACATTAATAAATTCTTTATCGAGAAACGGGACACGTGCTTCAACACCCCAAGCTGA
AGTTGCCTTGTTGGCTCTCAGGCAATCATACAGATGAAGAGCTTTAATCTTTCTGCATGTTTCCTGATGAAATTCTTCCTTGTTAGGTGCCTTAT
GGAAGTATAAGTAGCCACCAAAAATTTCATCAGATCCTTCACCAGAAAGAACCATTTTTACTCCCAGAGACTTGATTTTGCGAGACATCAGAA
ACATTGGGGTGCTGGCTCTGATAGTTGTAACATCATATGTTTCAATATGATATATAACCTCTTCCAGTGCATCTATGCCTTCCTGAACAGTGAA
ATAAAACTCATGGTGACGAGTTTGAAGGTAATCAGCTACCTCTCTGGCAGCTTTCAAATCTGGAGAACCCTTCAACCCAATGCAGAAGGAATG
CAACTGTGAACCCCACTGGTAAGCTCCCTCTGACTCAGCCAAGTGACGTGATGCCACAGCAGCAACAAGAGATGAATCCAGTCCGCCTGACA
GAAGAACACCAAACGGCACATCTGTCATTAGTCTCTTAACCACAGCCTTTTCAAAAGCTGCACGTAAAACTGAGGGATCATAGGGGGGTTGAA
GGAATCGTTTCTGAGTACCATGGTGGGTTGTACCACCTTCTTAGCGCTCCCTGTTTGCTTGAGTACATATGCCCAGGAGGAAAAGCGATGAAT
CGTTCACAGTCATCGCTTAGGGCCTTCATTTCCGAAGCAAACCAGACAGACCCATCAAGACCCCAGCCCATGTACAGTGGGGTAATACCAATA
GCATCACGAGCTGCTATGAAACTTTTATCACGGGTATCAAGAAGCACAAAGGAGAACATTCCATCCAGCATGTCCACAAAATCTTCTCCATGC

TCCTCATAAAGATGAGCAATCACTTCACAATCACTGCCAGTACGGAACTGATGTGACTTTAGATTCTCCCTTAGTTGTCTGTGGTTATATATTTC
TCCATTAACCGTTACAACAACTGTCTTGTCTTCATTGTAAAGTGGCTGATCTCCAGAAGTGGGATCTACAATAGCTAATCGCTGATGAGCGAG
ATAACAATTCTCAAAACAATGTAAGCCACTCCAATCAGGCCCTCTATGACGTAACCTTCGAGAAAGCTCAATGATCCTAGACCTCTTGGCCTGG
GAGTTATCGATACAGCCCAAGACCGCTAGTATTCCACACATGGCGGCCCTTCTTTCTGTGCTGCTGTAAACCTCTGCCTATCTCCTCTATTCTGG
CTATTGGGGACAGGAAACGCTGCAAAAAAGT

GDH_460

GGTAGAAACAGTTGGGGATTATAGCTTAGAAATTTGATCGATTATCACAATTCAATTTGACGATTTTGACGTCGAATTCAGAGGAGAGAGTGT
AATACTCATCTGGGAAAGAGGGCTTGTGAGGCTGGAGAGACCCGGAAAATCGGTTGTTGGAATTTTTTGGATTCCCGGATTTTTCCTCAGTTC
TTCAGAATCAGATATTCCAAAAGGTGGATAAGATTGACATGCTGAATGTTGCTTCCATTGAGTGGGTTAGGGATGAATTCTGCAATGGATGA
CATGAATCTGATTCAGCAGAGGCATCACTTGGTAGTCAGGGAGCTTGGAGAAGAAATTGACTTGGAAATTGGAGCTGGGGATGATGATCCA
TCATTTGCTAATGCCACTCTTATAAGTGGACCCCCTCGTGAGCCATCAGTGGAGGACAATGATGAGACAAAGCAAATGCAGATGGTGCCACC
CCTCCAGGGTGAAGAGCAGGATATGTCAAAGAGTCAGCCAACAAAGAAGAAGAAAAAAGTTGTGAAGCGGTGGAGAGAGGAATGGGCCG
ATACATACAAATGGGCATATGTTGATGTGAAAGACGGAACAGCAAGGATCTTTTGCTCTGTTTGCAGGGAGTATGGGAGGAAGCACAGAAG
GAATCCGTATGGTAATGAAGGTAGTAGAAACATGCAAATGAGTGCCTTGGAAGAACATAACAATAGCTTATTGCATAAAGAGGCTGTTCGGC
TGCAAATGGCGTCCAAGGATAAGATTGTTGTAGAGAAACCCATATATGTGAAAGCTCTGATGTCAAAAACAGCTGGATCAATTGTTGAGGTT
GCACTGAAAAGAGATCCTCACGAAGTTGAGTTTATTCAATCAGTGCAAGAAGCAGCGAACTCTTTAGAAAGAGTTATTGCGAAGAATTCTCAT
TATGTAAATATTATGGAACGTTTGCTTGAACCTGAGCGAACCATTATTTTCCGAGTTCCATGGGTTGATGATCGGGGTGAGACCCATGTTAAT
CGAGGCTTCCGGGTGCAGTTTAATCAGGCTTTAGGTCCATGTAGGGGAGGTGTTCGTTTCCATCCATCGATGAATCTAAGCATTGCAAAGTTT
CTTGCCTTTGAGCAGACTTTAAAGAATGCCTTATCTCCGTACAAGCTTGGAGGAGCTGCTGGTGGAAGTGATTTTGATCCAAAAGGGAAAAG
CGATAATGAGATCATGCGCTTTTGCCAAAGCTTTATGAATGAATTGTATCGGTATTTAGGACCAGATCAGGATCTACCTTCAGAGGAAATGGG
TGTTGGTGCTCGAGAGATGGGTTATCTTTTTGGGCAGTACAGGCGTCTTGCTGGTCACTTTCAGGGAAGTTTTACGGGACCAAGAATTTTCTG
GTCTGGATCTAGCCTACGAACAGAGGCCACTGGCTATGGGCTGGTCTTCTTTGCCCACCTTATTCTGGG

GDH_201910

GTTGGATGCTAGTTATATAAATTGAATGCTGATTCAGTGGAGTACATTGTTTTGTGGTGTATAATTGGCACAGTCATTATAATTTGGAACTTAC
AAACTCCTGCAAGAAGCAAGAGAAATTACCAAAAAAAACAATGTGAAACTTTCTAAAGGGGGGAGAACAAACTCCCCCCTGTCAACATAAAT
AAGCAACAGATCAAGGCAAAGGGTGACGACCCGGTGTGGACAATTTAATCTGTAAGGAAACCACAGAATGCACCTAAAACAAAGACAAAGG
AGGTGGTTTTTTTCTGTTGAAGAAGCCTAAATCACACCTCAACTCATGCTTCCCAACCTCTGAGAAGGGTTGCCCGAGCAACACGGTTAAGGC
CAAGGGTAAAGGCACCCATACGAAGATCACAATTGTGGGTCTGACACATAGCTTTCACGTCCTTGAGTCCCCGAGTCATGTATGTCTTGAGCT
CGGAGTTCACCCGATTTTCATCCCACATGAACCCCTGGATGTTCTGAACCCACTCAAAATAACTAACTGTAACTCCACCTGAGTTAGCATATAT
GTCAGGAAGGATGACCACGCCTTTCTTCCTCAAAATCTCATCAGCCTCTGGATCAGTTGGATGGTTAGCAGCCTCAACAATGAATTTGGCCTT
GATATCATTTGCATTGTCCCTGTTAATAACACCACCAAGAGCTGCTGGAATCAGGACATCGCAATCTTGAGAAAATATTGAGTTAGGATCTAT
GGCATCTGCACCGCTGAAATCTTTGACACCGCGAGTTTCTTGGACATGTTTAATTAGGCTGGGAATGTCAATCCCGTCCTTGTTCTTTATAGCT
CCGGTGATGTCACTTACAGCCACAACCTTCCCGCCCATTTCACTGATGAGTTGTGATGCCCAAGAGCCCACATTCCCAAAACCCTGTATCACAA
AGCGTTGTCCAGATATACTCTTTCCATACTCCTTCAGAAGGGCCTCTGTAGCAAAGAGCACTCCTCTTCCTGTAGCCGCATCTCTACCAAGAGA
TCCACCAAGGTCAATAGGTTTTCCAGTAACGACTGCAGGAGAATGACCATGAAATTTTGAGTACTCGTCCAATATCCAGGCCATTGTCTGTGC
ATTTGTCCCCATATCAGGTGCAGGAACATCAGTATGAATTCCAATGAGGTCATGAATCCTCTGAGTGAACACTCGGGTCAACCGCTCAAGCTC
AGAAGGACTTAAATCTGATGGGTTACAACCTATGCCACCCTTGGCACCACCATAAGGGATATTTGCCACTGCTGTCTTCCATGTCATTAGCTGA
GCCAATGCATTGACCTCATCTGGATCAACCTCTGGGTGGTATCTGATCCCGCCCTTCATAGGGCCTCTAGAGTTGTCATGCTGAACTCTAAATC
CAACATAAGATACGAGAGTGCCATCATCTTTGGGTATGGTGCACTCAACCTTGATCTCCCTAAATGGTATAAGAAGACTCCTTTCAAGTTTTGC
ATCCAACCCCAGGATCCGAGATGCGAGCTTAAAGTTCCTATTAGTTGCTGCAAGCGCATTCATGGTTTCCCACCTTAGAGTAAACAGAGAACG
GCAGCAAGGGATGAGGAATAGTTCCACTCAAATAGAAAGAATTACAAAAATGGGCTGAAATTAAAGAGGGTAAATGACAAATTGATTTGGG
GTTGAGCAGAGCACGGCAAAGCATAGCAGAAATTTCAGGAATTTATATAGAGAAGGGGCGGCCAAAGGAGGGGAACTGCAAGCACAAGGA
ATCAATTCTTTAATGGAATATTTAGCCTATGAAA

GS_30800

CCCCCCCCCCCCCTCTCTCTCTCTCTCGACTTCTTTCTCTCTCTTCCTGTTTGATTTGTGTTCGGTTGCTGAGGAAGCAGGGAAAAGTAACCAAA
CATGGCACAGATACTTGCACCTAACATGCAATGGCAGATGAAGATTACCAAAAGCTCAGCCCCTGGGAAATCAATGATATCAAGCTCAAGTG
CATGGACTTCTTTATTGATGAAACAAACCCGAAAAGGATCAATTAAGACAGTAACTAAATTTAAAGTGCGTGCTTCAGTCAAATCTGAAAATA
GTACTGTGAACAGGGTGGAGCAGCTACTCAATTTGGATGTCACTCCATACACTGACAAGATTATTGCTGAATACATTTGGATTGGAGGATCTG
GAATTGATCTCCGAAGCAAATCAAGGACAATCGCAAAACCTGTGGAGCACCCATCTGAGCTTCCCAAATGGAATTATGATGGATCTAGCACA
GGGCAGGCTCCAGGAGAAGACAGTGAAGTAATCTTATACCCACAAGCAATTTTTAAGGACCCATTCCGCGGTGGCAGCAACATTTTGGTAAT
TTGTGATACTTACACACCACAAGGTGAGCCAATCCCAACCAACAAACGGTACAAGGCTTCCCAGATCTTCAGTGATCCAAAGGTTGTTGCTGA
GATTCCATGGTATGGAATAGAGCAAGAATACACGTTGCTCCAACCAAATGTGAAGTGGCCCTTAGGTTGGCCTGTTGGAGCTTATCCTGGTCC
ACAGGGACCATATTACTGTGGTGTTGGAGCTGACAAATCTTTTGGGCGTGACATATCTGATGCTCACTACAAAGCATGCTTGTATGCTGGAAT
TAACATCAGTGGCACCAATGGGGAGGTCATGCCTGGTCAGTCGCTAATCGTGGTTGCTCGTGGGAATTCCAAGTGGGCCCAAGTGTTGGCAT
TGAAGCTGGAGATCATATTTGGGCTGCTAGATATATTCTTGAGAGAATTACTGAGCAAGCTGGGGTAGTTCTTACACTCGATCCAAAGCCAAT

TGAGGGTGATTGGAATGGTGCTGGGTGTCACACCAATTACAGCACCAAGAGCATGAGAGATGAGGGAGGCTACGAGGTAATCAAGAAGGC
GATTTTGAACTTATCATTGAGGCACAGAGAACATATTAGTGCATACGGAGAAGGGAATGAAAGAAGGTTGACTGGAAAGCACGAAACTGCC
AACATCAACACTTTCTCTTGGGGGTCGCTAATCGTGGTTGCTCCATTCGTGTGGGTCGGGATACAGAAAAGCAAGGAAAAGGCTATTTGGA
AGACAGGCGGCCAGCTTCAAACATGGACCCGTATGTGGTCACAGCTTTACTAGCCGAGACCACAATTCTATGGGAGCCAACTCTGGAGGCTG
AAGCACTTGCAGCCCAAAAACTTGCCCTCAACGTTTGACTACATCCCATGTATTGTTGACTTCCCTCGTTCGTGTTCTGATTTCTTCTGTGTTGG
GGATTGTGGAAGACTTTTCTTTTTCTTTTTAATTGATCATTTTGACGTTGGAAATTGTTGGAGTCATTTGGAGCAGAATGGACGACTTGGTCTT
TGTGTCTTCTCAAGTGGAAAGCAGCCTTAGTAAGCTGTCTTCTACCTTTGCTGGAAGTAGTAGGTGACTACTGTTCATTAAACCTAGAGCCTGA
CTGGAAGCAATGTCTCTAATTTAGAGGAGCTGCTGCATATATATCTATCCTCTTTCTTCAAGATGGAAGTTTTAGGGTCAGCGTGGTCTGATTT
ATTTCTATTATGGAACGTACTTGATAAAAGCTACTGTCAACAATTTGTTGGAGAAGATTGTCTCACAAATCAAATTTAATGGGACGCAATTTGT
GTGCGCT

GS_94700

CGATGCAGACCGGAAGAATCGAACACAGGTACTCCATCCCAAAGCACACTTACAAATGGAAATGCAAATTCAAGTTTCTCTTGAATTAACTTG
CACATTTTCTGCTGTAGGTTTGGCACTTTTCACGGCTTCCACAGGATGGTGGTGTTGGCGATCAAAGAAGTTACAACATATGGGTCCATGTTC
GAAGCTGGCCTCCTGTCCTCAAAGTATCCTTTCCCGTTCTGCTCTGTCTCTCGCCCAACGCGAATTGATGCACCTCGGTTAGCAACCCCCCATA
GAAAAGTCTCAATGTTGGCTGTTTCATGTCTTCCAGTGAGACGGCGCTCATTTCCTTCACCATAAGCAGCAATGTGCTCTTTGTGCCTCAATCC
AAGCTTTTCAATAGCCTTCTTAATCACCTCGTAGCCACCATCTTCCCTCATGGACTTGGTGCTGTAGTTTGTGTGCGCACCAGCACCATTCCAGT
CACCTGGAATGGGTTTTGGATCGAAGGAAAGCACCACTCCAGCAATCTCGGTGATCCTCTCCAAAATGTAACGAGCGATCCACAACTCATCTC
CAGCAGAAATTCCAACAGCAGGGCCAACTTGAAATTCCCACTGGCCTGGCATCACTTCTCCGTTAATTCCACTAATGTTAACTCCTGCATAGAG
GCAAGCCTTGTAGTGCGCATCAACAATGTCCCTTCCAAAGGCTTTGTCAGCACCAACACCACAATAATAGGGGCCCTGTGGGCCAGGGTAGC
CTCCGACAGGCCAGCCTAAAGGCCACTTAACATCCTTCTGAAGCAAAGTGTACTCTTGCTCGATTCCAAACCAAGGCTCCTCAGCAAGAACAT
CAGGGTGGCTGAATATCTTTTCGGCATTGTATCTCTTGTTTGTGGGGATGGGTTCTCCTTGTGGGGTGTATGCATCACACATTACAAGTATATT
GTTGCCCCTCCTGAATGGATCTCTGAAAATAGCTTGTGGGTAGAGGATAACTTCACTGTCTTCACCAGGAGCCTGACCAGTGCTAGATCCATC
ATAATTCCACTTGGGGAGCTTTTTGGGATCATCAACTGGTGCATTCAGAGTCCTTGCTTTGCTTCTCATGTCCATACCAGATCCACCAATCCATA
TGTACTCAGCAATCACCTTCTCGGTGCAATCAGAGAGATTAAGGTTAATGAGATCTGTAAGAAGAGACATAATTGGTAGATTCCTTGAGGAA
AGGAGAAGATACTGCCGAATTTGGGATCTCTCTGTTTGAATTTGGCGTTTAGAATCTGGGGAGATAACAGAGAGAGTCGTGAGGATGGGGC
AGAATCCTCTGGTTATTATATAGGAGTACAGAACAACAGAGCTACTATTGCCTTTTCTATCACTTTTTTTTTTTCTTTTTT

UBQ_19060

CCTCCCCTGAGACGGAGGACAAGCTGGAGAGTTGACTCCTTCTGGATGTTGTAGTCAGCAAGGGTGCGACCATCTTCAAGCTGTTTACCAGC
GAAGATCAACCTCTGCTGGTCTGGGGGAATGCCTTCCTTGTCTTGGATCTTGGCCTTGACATTATCAATAGTGTCGGAGCTCTCAACCTCCAAA
GTAATCGTCTTTCCTGTCAAAGTCTTCACAAAAATTTGCATGCCACCACGAAGACGAAGGACCAAGTGGAGGGTGGACTCCTTTTGGATGTTG
TAGTCAGCGAGAGTACGACCATCCTCAAGCTGTTTCCCAGCGAAGATCAATCGTTGCTGGTCTGGGGGAATTCCTTCCTTGTCTTGGATCTTG
GCCTTGACATTATCAATAGTGTCGGAGCTCTCAACCTCCAAAGTAATCGTCTTTCCTGTCAAAGTCTTCACAAAAATTTGCATGCCACCACGAA
GACGAAGGACCAAGTGGAGGGTGGACTCCTTTTGGATGTTGTAGTCAGCGAGAGTACGACCATCCTCAAGCTGTTTCCCAGCGAAGATCAAA
CGTTGCTGGTCTGGGGGAATTCCTTCCTTGTCTTGGATCTTCGCCTTAACGTTGTCAATGGTGTCTGAACTCTCGACCTCAAGGGTGATTGTCT
TGCCAGTAAGAGTCTTGACGAAGATTTGCATGCCCCCTCTGAGACGGAGGACAAGGTGAAGGGTGGACTCTTTCTGGATGTTGTAGTCAGCC
AGAGTCCTGCCATCCTCAAGCTGCTTGCCGGCAAAGATCAGCCTTTGCTGGTCTGGAGGGATTCCCTCCTTGTCTTGGATCTTGGCCTT

Actin_88560

GCCCAGAAAAATAGTAGTGTATGGATTCAAAACATTGGGCAAATGTTCCGCAAAGATTACATGCCATGACCCTTGAAAAATAGTTACAACAA
ACAATAGAAATTCCAAGCTTTTTGGTTCCCAAAAAAAAAAGAAAAAAAAAACAGACTCCTAAGTTTCTCATCTCATCTCAACCAAATCAAATCA
AATCATACAGCACACTAATAGTTTGACCAAACGGATAGAAAAATAAGGAGACAGCAGGGGACTATGTAGGACTAGGAACTCAAAAACTTATC
TTAGAAGCACTTCCGGTGAACAATTGATGGGCCAGACTCATCATATTCACCCTTGGAGATCCACATCTGTTGGAAGGTGCTGAGTGATGCCAG
GATAGACCCTCCTATCCAGACACTGTACTTCCTCTCAGGAGGAGCAACAACCTTGATCTTCATGCTGCTAGGAGCAAGGGCAGTAATTTCTTT
GCTCATACGGTCAGCAATACCCGGGAACATTGTTGTACCACCACTGAGCACAATGTTACCATAGAGATCCTTTCTGATATCGACATCACACTTC
ATGATGGAGTTGTAGGTTGTCTCGTGGATACCTGCGGCTTCCATACCAATCAATGAAGGCTGGAAGAGGACCTCTGGGCAACGGAACCTCTC
AGCTCCAATAGTGATCACCTGCCCATCAGGCAACTCATAGTTCTTTTCTACTGCAGAGCTGCTCTTGGCAGTCTCAGATTCTTGCTCAAAGTCA
AGAGCAACATAAGCAAGCTTCTCCTTAATATCACGGACAATTTCCCGTTCGGCTGATGTAGTGAACATGTAACCTCTCTCAGTGAGGATCTTCA
TTAGATAGTCGGTGAGATCACGACCAGCAAGATCCAAACGAAGGATGGCATGGGGAAGGGCGTAACCCTCATAGATCGGAACAGTGTGACT
CACACCATCACCAGAATCAAGCACAATACCTGTTGTACGACCACTGGCATAGAGAGAAAGAACAGCCTGGATGGCAACATACATAGCGGGCA
CATTGAATGTCTCAAACATGATCTGGGTCATTTTCTCTCTGTTAGCCTTTGGGTTCAAGGGGGCCTCTGTAAGCAACACTGGGTGCTCCTCTGG
AGCAACACGAAGTTCATTGTAAAAGGTGTGATGCCAGATCTTCTCCATGTCATCCCAGTTGCTTACAATACCATGCTCGATTGGGTACTTCAAG
GTCAAAATACCTCTTTTTGATTGAGCTTCATCTCCAACATAAGCGTCCTTCTGGCCCATACCAACCATGACACCAGTGTGCCTAGGACGACCGA
CAATACTTGGGAAAACAGCCCTTGGAGCATCATCTCCAGCAAACCCAGCCTTGACCATTCCAGTTCCATTGTCACAGACAAGGGGCTGGATAT
CCTCTCCATCAGCCATTTTAATTTATACTTGAAATCCGAAGACCTTGCGGCTGAGAGGAGAGAGAGAATGCAGGAGCAGAGAGAGAAAGCG
AGAGGCGAGAGAATGACAAGAGAGGCG

Supplemental Tables

Table S1: Recipes for nutrient working solutions that were stored in the dark at 4 °C and combined to make each treatment nutrient mix prior to application.

Reagent (pH 5.7-5.8)	Molar Mass (g·mol^{-1})	Stock Solution (mM)	g/500mL (Stock Solution)	Working Solution (mL/1.5L) [Final]	Working Solution (mL/1.5L) [Final]	Working Solution (mL/1.5L) [Final]
**$MgSO_4$ + 7H$_2$0**	246.47	500 mM	61.62	6.0 (2.0 mM)	6.0 (2.0 mM)	6.0 (2.0mM)
**KNO_3**	101.1	500 mM	25.28	2.52 (0.84 mM)	5.01 (1.67 mM)	10.02 (3.34 mM)
**$Ca(NO_3)_2$ + $4H_2O$**	236.15	500 mM	59.04	2.49 (0.83 mM)	4.98 (1.66 mM)	9.99 (3.33 mM)
**K_2SO_4**	174.26	500 mM	43.57	3.75 (1.25 mM)	2.52 (0.84 mM)	5.01 (1.67 mM)
**$(NH_4)_2H_2PO4$**	132.06	500 mM	33.02	3.75 (1.25 mM)	7.5 (2.5 mM)	15 (5.0 mM)
**$CaCl_2$**	110.98	500 mM	27.75	7.5 (2.5 mM)	5.01 (1.67 mM)	9.99 (3.33 mM)
**NaH_2PO_4**	120	500 mM	30	11.25 (3.75 mM)	7.5 (2.5 mM)	15 (5.0 mM)
**Micronutrients (pH 5.7-5.8)**		**Molar Mass (g·mol^{-1})**			**g/500ml**	
**H_3BO_3**		61.83			1.43	
**$MnCl_2$ + $4H_2O$**		197.91			0.91	
**$ZnSO_4$ + $7H_2O$**		287.56			0.11	
**$CuSO_4$ + $5H_2O$**		249.69.			0.04	
**H_2MoO_4**		164.92			0.01	
**Fe-EDTA (pH 5.7-5.8)**		**Molar Mass (g·mol^{-1})**			**g/250 mL**	
**Ethylenediaminetetraacetic acid (EDTA)**		292.24			6.5	
**$FeSO_4$ + $7H_2O$**		278.01			6.25	

Table S2: Amount of working solutions combined to make each treatment in ml. The resulting nutrient solution was brought to a final volume of 1.5 L with distilled water. 250 ml of the final solution was added to each of six individual *O. ficus-indica* plants in each treatment twice a week for one month after.

	**0.0 mM NO_3^- + 0.0 mM NH_4^+**	**2.5 mM NO_3^- + 0.0 mM NH_4^+**	**2.5 mM NO_3^- + 0.0 mM NH_4^+**	**10.0 mM NO_3^- + 0.0 mM NH_4^+**	**10.0 mM NO_3^- + 10.0 mM NH_4^+**	**0.0 mM NO_3^- + 2.5 mM NH_4^+**	**0.0 mM NO_3^- + 5.0 mM NH_4^+**	**0.0 mM NO_3^- + 10.0 mM NH_4^+**	**2.5 mM NO_3^- + 2.5 mM NH_4^+**	**2.5 mM NO_3^- + 5.0 mM NH_4^+**	**2.5 mM NO_3^- + 10.0 mM NH_4^+**	**10.0 mM NO_3^- + 2.5 mM NH_4^+**	**5.0 mM NO_3^- + 2.5 mM NH_4^+**	**5.0 mM NO_3^- + 5.0 mM NH_4^+**	**5.0 mM NO_3^- + 10.0 mM NH_4^+**	**10.0 mM NO_3^- + 5.0 mM NH_4^+**	**diH_2O**
**$MgSO_4$**	6	6	6	6	6	6	5	6	6	6	6	6	6	6	6	6	0
**KNO_3**	0	2.52	5.01	10.02	10.02	0	0	0	2.52	2.52	2.5	10.02	5.01	5.01	5.01	10.02	0
**$Ca(NO_3)_2$**	0	2.49	4.98	9.99	9.99	0	0	0	2.49	2.49	2.49	9.99	4.98	4.98	4.98	9.99	0
**K_2SO_4**	5.01	3.75	2.52	0	0	5.01	5.01	5.01	3.75	3.75	3.75	0	2.52	2.52	2.52	0	0
**$CaCl_2$**	9.99	7.5	5.01	0	0	9.99	9.99	9.99	7.5	7.5	7.5	0	5.01	5.01	5.01	0	0
**NaH_2PO_4**	15	15	15	15	0	11.25	7.5	0	11.25	7.5	0	11.25	11.25	7.5	0	7.5	0
**$(NH_4)_2H_2PO4$**	0	0	0	0	15	7.5	7.5	0	3.75	7.5	15	3.75	3.75	7.5	15	7.5	0
**Micronutrients**	1.5	1.5	1.5	1.5	1.5	1.5	1.5	1.5	1.5	1.5	1.5	1.5	1.5	1.5	1.5	1.5	0
**Fe-EDTA**	1.5	1.5	1.5	1.5	1.5	1.5	1.5	1.5	1.5	1.5	1.5	1.5	1.5	1.5	1.5	1.5	0

Table S3: List of primers designed for RT-qPCR analysis with predicted subcellular localizations of the gene products.

Gene name	Gene/ primer name	Gene description	Subcellular localization	Primer sequence (F, forward; R, reverse)	Annealing temperature (°C)	Amplicon length (bp)
Op_fin206820	ALMT	Aluminum-activated malate transporter	Tonoplast (Mitochondria)	F 5′ TTTCCTATCTGGGCTGGC 3′<br>R 5′ GAAGCAGCAACACCCTTGAAA 3′	54.5 °C<br>56.2 °C	68
Op_fin7190	PPC	Phosphoenolpyruvate carboxylase	Cytosol	F 5′ AGGCACAATGATGTGATGGA 3′<br>R 5′ TTCTTCAGTTTTGGGGAGATTGG 3′	55.3 °C<br>55.2 °C	150
Op_fin211860	PEPCK	Phosphoenolpyruvate carboxykinase (ATP-like)	Cytosol	F 5′ AACCAGTGTTGACGAGCCA 3′<br>R 5′ GCTGGTACGGTAGATGGCAT 3′	56.9 °C<br>56.9 °C	154
Op_fin52570	NR	Nitrate reductase	Cytosol	F 5′ CCACACTTTGACCCTGTCC 3′<br>R 5′ TACGCCAATCGGACAGAGGA 3′	55.8 °C<br>58 °C	87
Op_fin241390	NiR	Nitrite reductase	Chloroplast	F 5′ ACAATAGGCACCAAGTCC 3′<br>R 5′ ATATCGGTTTCATGGGTTGC 3′	52.1 °C<br>53.1 °C	151
Op_fin81140	GOGAT	Glutamate synthase	Chloroplast	F 5′ GTAAAGTTGAACCTGCTTCATCTA 3′<br>R 5′ GATTTTCCACCCAATCTGTTC 3′	52.9 °C<br>51.8 °C	124
Op_fin236590	AS	Asparagine synthase	Cytosol	F 5′ ATCCCACTCCGTCACTGAAC 3′<br>R 5′ CGCAATGCCTTTGATGATGAT 3′	56.8 °C<br>54.1 °C	88
Op_fin460	GDH	Glutamate dehydrogenase	Cytosol	F 5′ AGTGGGTTAGGGATGAATT 3′<br>R 5′ TCCAATTTCCAAGTCAATTTCTT 3′	50.6 °C<br>51.0 °C	105
Op_fin201910	GDH	Glutamate dehydrogenase	Cytosol	F 5′ ACCAAGAGATCCACCAAGG 3′<br>R 5′GCACCTGATATGGGGACAAA 3′	54.2 °C<br>54.9 °C	123
Op_fin30800	GS	Glutamine synthetase	Chloroplast	F 5′ GGAATGAAAGAAGGTTGACTGGA 3′<br>R 5′ GAGCAACCACGATTAGCGAC 3′	55.0 °C<br>56.2 °C	82
Op_fin94700	GS	Glutamine synthetase	Cytosol	F 5′AATCACCTCGTAGCCACC 3′<br>R 5′ GATTGCTGGAGTGGTGCTT 3′	54.4 °C<br>55.6 °C	121
Op_fin19060	UBQ	Ubiquitin	Cytosol (Mitochondria)	F 5′ CTTCTGGATGTTGTAGTCAGC 3′<br>R 5′ ACTTTGGAGGTTGAGAGC 3′	53.1 °C<br>51.9 °C	150
Op_fin88560	Actin	Actin	Cytosol	F 5′ CACATCTGTTGGAAGGTGC 3′<br>R 5′ CTGACCGTATGAGCAAAGAAAT 3′	53.9 °C<br>53.1 °C	139
